# Exposure to sugar rationing in first 1000 days after conception and long term cardiovascular outcomes: natural experiment study

**DOI:** 10.1136/bmj-2024-083890

**Published:** 2025-10-22

**Authors:** Jiazhen Zheng, Zhen Zhou, Jinghan Huang, Qiang Tu, Haisheng Wu, Quan Yang, Peng Qiu, Wenbo Huang, Junchun Shen, Chuang Yang, Gregory Y H Lip

**Affiliations:** 1 Bioscience and Biomedical Engineering Thrust, Systems Hub, The Hong Kong University of Science and Technology (Guangzhou), Guangzhou, Guangdong, China; 2 School of Public Health and Preventive Medicine, Monash University, Melbourne, VIC, Australia; 3 Biomedical Genetics Section, School of Medicine, Boston University, Boston, MA, USA; 4 Department of Chemical Pathology, Faculty of Medicine, Chinese University of Hong Kong, Hong Kong Special Administrative Region; 5 Faculty of Medicine and Health, University of Sydney, Australia; 6 School of Public Health, LKS Faculty of Medicine, University of Hong Kong, Hong Kong Special Administrative Region; 7 Cardiac and Vascular Center, University of Hong Kong-Shenzhen Hospital, Shenzhen, China; 8 Department of Vascular Surgery, Shanghai Ninth People's Hospital, Shanghai JiaoTong University School of Medicine, Shanghai, China; 9 Department of Clinical Epidemiology and Health Economics, University of Tokyo, Tokyo, Japan; 10 Department of Visceral, Transplant, Thoracic and Vascular Surgery, University Hospital Leipzig, Leipzig, Germany; 11 Liverpool Centre for Cardiovascular Science, University of Liverpool, Liverpool John Moores University, Liverpool Heart and Chest Hospital, Liverpool, UK; 12 Department of Clinical Medicine, Aalborg University, Aalborg, Denmark; 13 Medical University of Bialystok, Bialystok, Poland

## Abstract

**Objective:**

To examine whether exposure to sugar rationing during early life is associated with a reduction in the risk of cardiovascular outcomes in adulthood.

**Design:**

Natural experiment study.

**Setting:**

UK population based cohort.

**Participants:**

63 433 UK Biobank participants born between October 1951 and March 1956 without prevalent cardiovascular disease, multiple births, adoption, or birth outside the UK. Exposure was quasi-experimentally assigned on the basis of birth date relative to the end of sugar rationing in 1953. External validation cohorts from the Health and Retirement Study and the English Longitudinal Study of Ageing were used.

**Main outcome measures:**

Primary outcomes were incident cardiovascular disease, myocardial infarction, heart failure, atrial fibrillation, stroke, and cardiovascular disease mortality, ascertained through linked health records. Hazard ratios were estimated using Cox and parametric hazard models adjusted for demographic, socioeconomic, lifestyle, parental health, and genetic factors and geographical controls. Multiple cardiac parameters were measured in a subset undergoing cardiac magnetic resonance imaging.

**Results:**

Longer exposure to sugar rationing was associated with progressively lower cardiovascular risks in adulthood. Compared with people never exposed to rationing, those exposed in utero plus 1-2 years had hazard ratios of 0.80 (95% confidence interval (CI) 0.73 to 0.90) for cardiovascular disease, 0.75 (0.63 to 0.90) for myocardial infarction, 0.74 (0.59 to 0.95) for heart failure, 0.76 (0.66 to 0.92) for atrial fibrillation, 0.69 (0.53 to 0.89) for stroke, and 0.73 (0.54 to 0.98) for cardiovascular disease mortality. Incident diabetes and hypertension jointly mediated 31.1% of the sugar rationing-cardiovascular disease association, whereas birth weight contributed only 2.2%. Sugar rationing was also associated with a modest increase in left ventricular stroke volume index (0.73 (95% CI 0.05 to 1.41) mL/m^2^) and ejection fraction (0.84%, 95% CI 0.40% to 1.28%).

**Conclusion:**

Exposure to sugar rationing during the first 1000 days of life was associated with lower cardiovascular risks in adulthood and slightly more favourable cardiac indices, suggesting long term cardiovascular benefits of early life sugar restriction.

## Introduction

As noted in the United Nations’ 2030 Agenda for Sustainable Development,^1^ high sugar diets have become entrenched in global food cultures. With policy debates centring on sugar taxes,^2^ limits on added sugars in infant foods,^3^ and regulation of related marketing, establishing whether early life exposure to dietary sugars is associated with risk of chronic disease risk in later life is critical.

Accumulating evidence suggest that the first 1000 days (conception to ~2 years of age) is a period with heightened biological susceptibility,^4^ during which external factors including dietary patterns, pathogenic exposures, and socioeconomic conditions exert profound and lasting effects on predisposition to disease.^5^ The maturation of metabolic and cardiovascular systems during the first 1000 days shows exceptional plasticity,^6^ with their developmental trajectories being markedly responsive to nutritional inputs,^7^ endocrine signals, and broader environmental conditions. Moreover, nutritional interventions in the first 1000 days was shown to yield greater cost efficiency and long term health benefits than managing non-communicable diseases in adulthood.^8^ Furthermore, current World Health Organization guidelines emphasise optimal infant feeding practices, advocating exclusive breastfeeding during the initial six months of life followed by sustained breastfeeding with appropriate complementary foods until 24 months of age.^9^

On average, pregnant and breastfeeding women consume more than 80 g of added sugars daily,^10^ three times the recommended amount,^11^ raising concerns about the potential exposure to an adverse intrauterine environment for the fetus. Although breast milk is typically free from added sugars and the glucose content in breast milk is not significantly influenced by maternal diet,^12^ as children are gradually introduced to solid foods during weaning, they may become exposed to added sugars found in processed foods.^13^ ^14^ Unlike breast milk, which is naturally sugar-free, commercial baby foods, infant formulas, and other grocery items often consumed by infants may contain sucrose and other added sugars.^15^ A sampling survey found that 74% of baby foods tested contained ≥20% of total calories from added sugars per serving.^16^ Many such products are marketed to infants and often contain sugar levels higher than those indicated on nutrition labels, exceeding the recommended daily intake for infancy.^16^

The “fetal origins of disease” hypothesis in the cardiovascular domain was supported by animal studies linking early overexposure to sugar to endothelial dysfunction, vascular remodelling, and persistent cardiac alterations.^17^ ^18^ A high sucrose diet in pregnant mice was reported to lead to fetal programming that resulted in cardiometabolic diseases in offspring, with male offspring showing cardiac arrhythmias and altered heart rate variability.^19^ Furthermore, the intake of liquid fructose by rats during pregnancy was shown to affect the expression of cardiac genes related to osmotic pressure.^20^

Recent human studies have indicated that maternal metabolic conditions are associated with changes in the offspring’s cardiac health and parameters from an early age. For instance, Gertler and Gracner found that early life sugar restriction was associated with a lower prevalence of elevated cholesterol, cardiovascular disease and other comorbidities, and chronic inflammation.^21^ In addition, a study found that children of mothers in the highest quarter of one hour oral glucose tolerance test values, compared with those in the lowest quarter, showed a lower left ventricular ejection fraction (−1.8%) and 58% greater odds of having elevated systolic blood pressure (≥90th centile).^22^ In a retrospective study of 19 171 mother-child pairs, high maternal sugar concentrations were linked to a higher risk of congenital heart disease in offspring.^23^ Another study found that offspring of mothers with obesity had persistently lower left ventricular strain (−2.4 during fetal life and up to −0.4 in infancy) and thicker interventricular septa (0.6 mm).^24^ However, the long term effects of sugar rationing on cardiovascular outcomes in later life remain unclear.

We leveraged a natural experiment based on the UK’s sugar rationing policy, introduced in July 1942 as part of a broader 14 year wartime food rationing programme aimed at ensuring equitable food distribution and preventing shortages and famine during and after the second world war.^25^ This system relied on scientifically calculated weekly allowances to maintain the minimum nutritional intake needed for health, with sugar and sweets being strictly limited. During sugar rationing, each person, including pregnant women and children aged 5 and above, received approximately 8 ounces of sugar weekly and 12 ounces of sweets monthly through a ration book system registered with designated retailers.^11^ ^25^ ^26^ Notably, children under 2 years of age were not allocated sugar or sweets as part of the ration.^27^ The rationing system curtailed sugar intake to levels consistent with current dietary guidelines; specifically, adults consumed less than 40 g of sugar per day and children under 5 consumed less than 15 g per day.^11^ A previous study indicated that after the end of sugar rationing in September 1953, a sharp increase in the consumption of sugar and sweets occurred, suggesting a significant surge in their consumption patterns.^28^ Specifically, Gracner and colleagues showed that the average daily sugar consumption for an adult markedly increased after the end of sugar rationing in September 1953, escalating from 41 g during the first quarter of 1953 to around 80 g by the third quarter of 1954. Significantly, the complete termination of rationing occurred in July 1954, whereas the intake of other foods and nutrients, barring sugar, either stayed constant or showed minor changes during this timeframe.^29^ This led to quasi-experimental changes in early sugar intake, providing an exceptional opportunity to assess the long term health effects of constrained sugar exposure during critical developmental periods.

In this study, we estimated the long term effects of sugar rationing during the first 1000 days after conception on risks of cardiovascular outcomes in adulthood. We assessed the risks of multiple cardiovascular outcomes—cardiovascular disease, myocardial infarction, heart failure, atrial fibrillation, stroke, and cardiovascular disease mortality—by comparing individuals exposed to sugar rationing in utero and infancy under the rationing system with those exposed to higher sugar levels after rationing ended. We hypothesised that sugar rationing during the first 1000 days reduced the risks of cardiovascular outcomes and delayed their onset and that longer durations of constrained exposure provided progressively greater protection. Additionally, we incorporated cardiac magnetic resonance imaging (MRI) indices to explore subclinical cardiac alterations. Gracner and colleagues used the same natural experiment in their study and found that early life rationing reduced the risk of diabetes and hypertension by approximately 35% and 20%, respectively.^29^ As these two diseases are risk factors for cardiovascular disease, we further did a mediation analysis to assess how diabetes, hypertension, and birth weight may explain the link between early life sugar rationing and long term cardiovascular risk.

## Methods

### Study design and participants

**Fig 1:**
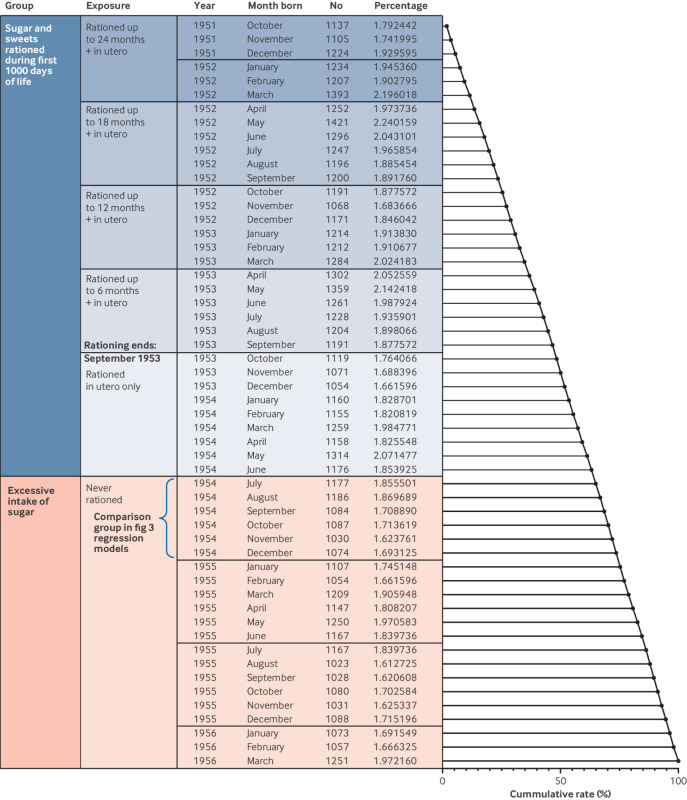
Sample distribution of births by calendar months and exposure to sugar rationing. Sugar rationed group is represented in blue; group that was never exposed to sugar rationing is represented in orange. First group of those never exposed to sugar rationing is labelled and was used as control group to assess association of early life rationing exposure with cardiovascular outcomes

We used an event study approach to examine the long term effects on cardiac health of limited sugar exposure during the first 1000 days after conception. We used exposure to national sugar rationing policies as a proxy for sugar intake in early life. Specifically, we used the end of sugar rationing in September 1953, which triggered a sharp increase in sugar consumption but did not substantially affect other food types,^28^ ^29^ as a natural experiment. This allowed us to compare adults who were exposed to sugar rationing in early life with those who were not. Birth year determined whether individuals experienced sugar rationing in early life, quasi-experimentally assigning them to either the rationed (low sugar) or non-rationed (high sugar) groups during pregnancy or early childhood. Figure 1 illustrates the timeline of sugar rationing and categorises individuals into rationed and non-rationed groups on the basis of their birth date. For this quasi-experimental design, which simulates sugar rationing and was previously used by Gracner and colleagues,^29^ the details on how the 1000 day window around the end of rationing was defined and the rationale behind the regression discontinuity design used in the study can be found in Gracner and colleagues’ study.^29^

The National Food Survey (NSF) was used to examine quarterly dietary patterns during and after the period of rationing from 1950 to 1960. The NSF collected weekly dietary records from a representative panel of more than 10 000 households, providing detailed data on dietary habits, nutrient intake, and economic trends that inform food policy and public health strategies.^30^ Detailed yearly data on food intake, the Food Price Index, the All Items Consumer Price Index, and other nutritional and socioeconomic indicators relevant to this study can be found in Gracner and colleagues’ study^29^ or downloaded directly from https://www.gov.uk/government/statistics/family-food-historic-reports. As a supplement, we plotted trends in sugar consumption over time across different socioeconomic strata (supplementary figure A).

The goals, participant demographics, and data collection methods of the UK Biobank study have been previously documented.^31^ In brief, between 2006 and 2010, the UK Biobank study recruited more than 0.5 million participants, aged 40-70, from the general population at 22 assessment centres across England, Scotland, and Wales. Data collection involved questionnaires, interviews, regular assessment centre visits, and health record linkages, covering diverse psychosocial, sociodemographic, physical, and genetic variables.^31^

**Fig 2:**
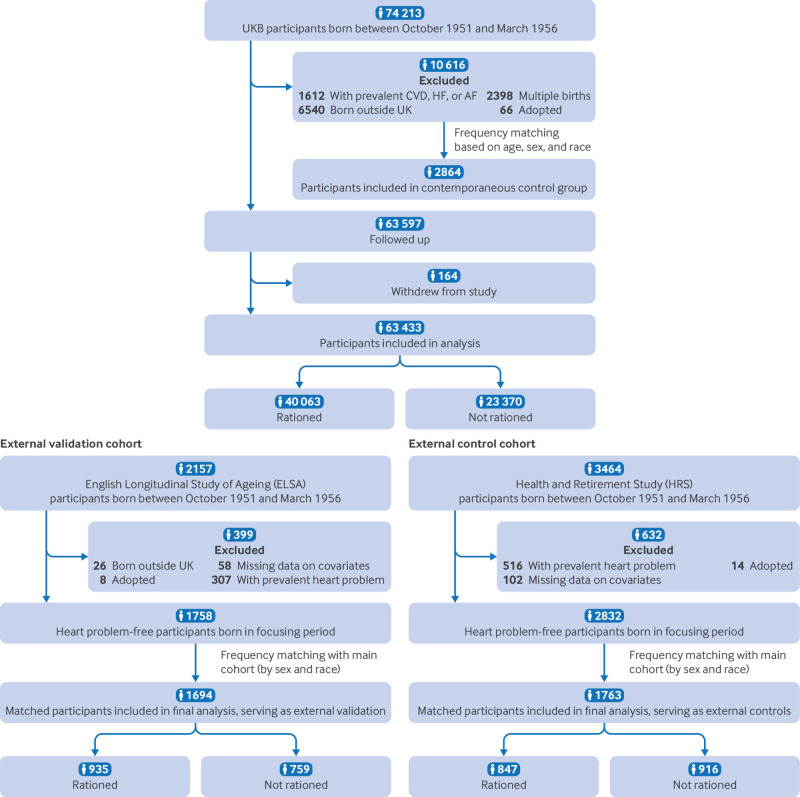
Flowchart of study cohort. AF=atrial fibrillation; CVD=cardiovascular disease; HF=heart failure; UKB=UK Biobank

Of 74 213 UK Biobank participants born between October 1951 and March 1956, we excluded 10 616 participants because of prevalent cardiovascular disease, heart failure, or atrial fibrillation (n=1612); being born outside the UK (n=6540); being from multiple births (n=2398); or being adopted (n=66). After exclusion of 164 participants who withdrew, 63 433 participants remained for analysis, with 40 063 exposed to sugar rationing and 23 370 not exposed **(** fig 2 **)**.

### Early life exposure to sugar rationing and covariates

We grouped participants on the basis of the length of their exposure to sugar rationing in early life (fig 1). To improve statistical power in the main analysis, we further combined these into in utero only, in utero plus up to one or two years, or never exposed.

At the baseline assessment (2006-10), participants completed a touch screen questionnaire that collected variables including gender, age, ethnicity, place of birth (England, Wales, Scotland, or outside the UK), household income, education level, Townsend deprivation index, smoking, alcohol consumption, physical activity, parents’ health conditions, and early life factors (birth weight, maternal smoking around birth, and whether the participant was breastfed as a baby). The place of birth within the UK (north and east coordinates) and birth weight were obtained through interviews conducted by trained researchers. Digestive diseases (that is, diseases related to the gastrointestinal tract), kidney diseases, and liver diseases were identified using ICD-10 (international classification of diseases, 10th revision) codes.

Participants were classified as having hypertension if they met any of the following criteria: use of antihypertensive drugs, systolic blood pressure >140 mm Hg, diastolic blood pressure >90 mm Hg, or self-reported hypertension. High cholesterol was defined by a self-reported diagnosis, the use of lipid lowering drugs, or a serum total cholesterol concentration of ≥200 mg/dL. Diabetes was defined by a self-reported diagnosis, the use of antidiabetes drugs, or a haemoglobin A_1c_≥6.5% or fasting blood glucose ≥126 mg/dL. Detailed information on the calculation of the polygenic risk score is provided in the supplementary methods. For more detailed information on these measurements, please visit the UK Biobank website (www.ukbiobank.ac.uk).

### Assessment of cardiovascular outcomes and placebo outcomes

In this study, the primary outcomes were cardiovascular disease, myocardial infarction, heart failure, atrial fibrillation, stroke, and cardiovascular disease mortality. We obtained the dates and causes of death by linking to the death registries of the NHS Information Centre for England and Wales, as well as the NHS Central Register for Scotland. Additionally, we identified the dates and causes of hospital admissions through linkage to the Scottish Morbidity Records for Scottish participants and the Hospital Episode Statistics for participants from England and Wales.^31^ We defined cardiovascular disease by ICD-10, using codes I20-I25 and I60-I64; myocardial infarction by codes I21, I22, I23, I24.1, or I25.2; heart failure by code I50; atrial fibrillation by code I48; stroke by codes I60-I64; and cardiovascular disease death by codes I00-I99. Each outcome referred to the first event recorded for each individual.

As placebo outcomes, we selected osteoarthritis and cataract because they are common in older adults and have no known biological link to early life sugar exposure. Although osteoarthritis has been increasingly linked to metabolic health,^32^ previous studies indicate that its primary risk factors are genetic predisposition, mechanical loading, and ageing,^33^ whereas the direct impact from glucose metabolism seems to be limited.^34^ ^35^ We defined these conditions by using ICD-10 codes M15-M19 for osteoarthritis and H25-H26 for cataract. The follow-up period for all participants started at recruitment and ended at the time of outcome diagnosis, death, loss to follow-up, or the study’s end (1 July 2023), whichever occurred first.

### Cardiac magnetic resonance imaging

Among the approximately 500 000 initial UK Biobank participants, four imaging assessment centres conducted imaging enhancement studies on participants within feasible travel distances. As of August 2023, more than 48 000 participants had completed these imaging studies. The MRI scans considered in this study (>6500 scans) were performed an average of 8.8 (standard deviation 1.6) years after the initial visit. The method for cardiac MRI acquisition in the UK Biobank has been described before (supplementary methods).^36^

Cardiac MRI segmentation and analysis were done using a certified deep learning algorithm, which helped in obtaining cardiac metrics. Where necessary, these phenotypes were adjusted for body surface area. The cardiac phenotypes derived were left ventricular stroke volume index, left ventricular mass index, left ventricular end diastolic volume index, left ventricular mass-to-volume ratio, and left ventricular ejection fraction.

### Contemporaneous validation and control group

We additionally analysed non-UK born adults in the UK Biobank and the Health and Retirement Study^37^ (HRS) as supplementary negative controls. Because these populations did not experience sugar rationing or similar policy changes around 1953, null associations by birth cohort are expected. The aim of these analyses is not to provide a direct counterfactual for the UK born group, but rather to help to rule out the possibility that global secular trends, measurement artefacts, or sample processing biases could explain our findings. In the UK Biobank, after frequency matching with the UK born group on age, sex, and race, we included 2864 participants from the contemporaneous control group who met the matching criteria.

In addition, we used the English Longitudinal Study of Ageing (ELSA)^38^ as contemporaneous external validation. HRS and ELSA examined ageing populations in the US and the UK. Both longitudinal studies use biennial assessments through standardised questionnaires and comparable measurement instruments to evaluate participants’ economic conditions, physical health, and psychological well being across time. Data from the HRS spans waves 4-12 (1998-2014), and the ELSA encompasses waves 1-9 (2002-18). For analytical purposes, wave 4 of HRS (1998) and wave 1 of ELSA (2002) have been designated as baseline measurements in their respective studies. HRS and ELSA identified outcomes through biennial surveys. To harmonise outcome definitions, we used the broad, self-reported measure of “ever had heart problems,” based on whether the participant had ever had any heart condition diagnosed by a doctor. This unified definition accommodated differences in questionnaire phrasing across studies: ELSA provided more specific heart disease types (for example, angina, myocardial infarction), whereas HRS used a general question. Given the limited sample size for specific diagnoses, we applied this broader outcome for comparability. For estimation, we used Cox proportional hazards models in both datasets to evaluate the association between early life rationing exposure and risk of heart problems. For validation purposes, we used a simplified adjustment model, which included age, sex, race, education, marital status, and survey year. The follow-up period for all participants began at recruitment and ended at the time of outcome reported, death, loss to follow-up, or study completion, whichever occurred first. More details about the HRS and ELSA cohorts can be found in the supplementary methods.

The validation group, born during the same period as the study population in the UK, serves to test whether sugar rationing is associated with risk of heart problems in this population. After frequency matching with the UK born group on sex and race, we included 1694 participants from the ELSA and 1763 from the HRS. Figure 1 shows the inclusion and exclusion criteria for ELSA and HRS.

### Statistical analysis

Detailed information on the statistical analysis is provided in the supplementary methods. Continuous variables are presented as mean (standard deviation) and median (interquartile range), and categorical variables are presented as numbers (percentages). Missing values are shown in supplementary table A. To minimise inferential bias, we used multiple imputation by chained equations on 20 datasets,^39^ with detailed processes shown in the supplementary methods. We used χ^2^ tests to determine the P value for categorical variables and Mann-Whitney U tests for continuous variables, to assess differences between the rationed and non-rationed groups. We used Cox proportional hazards model and parametric hazard models based on the Gompertz distribution to estimate hazard ratios and corresponding 95% confidence intervals that describe the associations between sugar rationing and the incidence of cardiovascular outcomes. We evaluated the proportional hazards assumption by using a Schoenfeld residuals plot, and we detected no deviation from the assumption in this study.^40^ We chose the Gompertz distribution after assessing the best fitted distribution by using the Akaike and bayesian information criteria (supplementary table B).^41^

We used a directed acyclic graph to guide covariate selection (supplementary figure B). Model 1 adjusted for age and sex only. Model 2 incorporated directed acyclic graph selected covariates, which excluded post-treatment (that is, adult level) variables and included age, sex, race, birth location, calendar month of birth, real food prices (adjusted for the Consumer Price Index), genetic risk score for cardiovascular outcomes, parental history of cardiovascular disease, diabetes, or hypertension, maternal smoking around birth, breastfeeding status, and survey year. Model 3 used the same covariates as model 2 but used a parametric hazard model based on the Gompertz distribution. As a sensitivity analysis, we additionally adjusted for later life lifestyle and health related factors. In the subgroup analysis, we assessed potential modification effects on the basis of several factors, including sex (male, female), ethnicity (white, non-white), place of birth (England, Wales/Scotland), and polygenic risk score for cardiovascular disease, myocardial infarction, heart failure, atrial fibrillation, or stroke (low, medium, high). Additionally, we considered whether parents had a diagnosis of cardiovascular disease, diabetes, or hypertension (yes, no).

To account for potential concerns about general trends or spurious correlations affecting our results, we re-estimated the full model for the placebo outcomes of osteoarthritis and cataract. We used ordinary least squares to evaluate the relation between exposure to sugar rationing and cardiac MRI indices, including left ventricular stroke volume index, left ventricular mass index, left ventricular end diastolic volume index, left ventricular mass-to-volume ratio, and left ventricular ejection fraction, with models 1-2 following the same adjustments as mentioned above. We used a Fine and Gray model to adjust for competing risks, with non-cardiovascular disease mortality as the competing event.^42^ In addition, we used time-to-event models assuming Gompertz distribution to estimate the effect of rationing on the delay in age of disease onset.^41^ We additionally evaluated the association between sugar rationing and all cause mortality, using the same set of covariates in model 3. We applied a standard mediation analysis (Kenny and Baron 4 step analysis)^43^ to investigate the proportion mediated by diabetes, hypertension, and birth weight in the relation between sugar rationing and cardiovascular disease. The detailed steps are shown in the supplementary methods. We used R version 4.0.2 for all statistical analyses, with a significance level set at P<0.05 (two sided). For baseline characteristics and subgroup analysis, we have applied Bonferroni correction to our P values. The adjusted significance thresholds for these tests are 0.05/12=0.004 and 0.05/42=0.0012, respectively.

### Patient and public involvement

No funding was available for patient or public involvement in this project. The UK Biobank resource included extensive public consultation in its design. No patients were involved in setting the research question or the outcome measures, or in developing plans for design or implementation of the study. No patients were asked to advise on interpretation or writing up of results.

## Results

### Participants’ characteristics

The study included 63 433 participants (mean age 54.6 (standard deviation 1.6) years), of whom 40 063 were classified as the rationed group and 23 370 as the non-rationed group (table 1). The rationed group was older (55.4 *v* 53.2 years) and had a higher proportion born between March and May (29.3% *v* 20.8%) and a greater prevalence of parental history of cardiovascular disease (58.5% *v* 56.5%). Additionally, parents of the rationed group participants were less likely to have diabetes or hypertension or still be alive (13.4% *v* 20.1%). For adult level variables, the rationed group included fewer people with household incomes over £100 000 (2680 (6.7%) *v* 1817 (7.8%)), had a lower Townsend deprivation index (−1.5 *v* −1.4), and included fewer current smokers (4276 (10.7%) *v* 2755 (11.8%)) and more people with comorbidities (supplementary table C). Density distributions of polygenic risk score for various cardiovascular outcomes are similar between the rationed and not rationed groups (supplementary figure C).

**Table 1:** Baseline characteristics of participants born between October 1951 and March 1956. Values are numbers (percentages) unless stated otherwise

Characteristics	Total (n=63 433)	Rationed (n=40 063)	Not rationed (n=23 370)	Difference (percentage points)	P value*
Mean (SD) age at entry, years	54.6 (1.6)	55.4 (1.2)	53.2 (1.0)	2.2	<0.001
Women	36 096 (56.9)	22 777 (56.9)	13 319 (57.0)	−0.1	0.74
Place of birth:					
England	54 581 (86.0)	34 563 (86.3)	20 018 (85.7)	0.6	0.004
Wales	3096 (4.9)	1979 (4.9)	1117 (4.8)	0.1	
Scotland	5756 (9.1)	3521 (8.8)	2235 (9.6)	−0.8	
Birth month:					
1 Mar to 31 May	16 599 (26.2)	11 742 (29.3)	4857 (20.8)	8.5	<0.001
1 Jun to 31 Aug	14 328 (22.6)	8608 (21.5)	5720 (24.5)	−3.0	
1 Sep to 30 Nov	15 422 (24.3)	9082 (22.7)	6340 (27.1)	−4.4	
1 Dec to 28 Feb	17 084 (26.9)	10 631 (26.5)	6453 (27.6)	−1.1	
White	61 029 (96.2)	38 612 (96.4)	22 417 (95.9)	0.5	0.004
Parents’ condition:					
Diagnosis of cardiovascular disease†	36 634 (57.8)	23 425 (58.5)	13 209 (56.5)	2.0	0.005
Diagnosis of diabetes†	11 737 (18.5)	7246 (18.1)	4491 (19.2)	−1.1	0.01
Diagnosis of hypertension†	29 297 (46.2)	18 077 (45.1)	11 220 (48.0)	−2.9	<0.001
Still alive	10 070 (15.9)	5373 (13.4)	4697 (20.1)	−6.7	<0.001
Mean (SD) birth weight, kg‡	3.3 (0.5)	3.3 (0.5)	3.3 (0.5)	0.02	0.57
Maternal smoking around birth	19 354 (30.5)	12 216 (30.5)	7138 (30.5)	−0.0005	0.90
Breastfed as baby	37 165 (58.6)	23 564 (58.8)	13 601 (58.2)	0.6	0.13

SD=standard deviation.

* P values were obtained from either χ^2^ test or Mann-Whitney U test comparing difference between rationed and not rationed group.

† Mantel-Haenszel χ^2^ test applied to adjust for whether participants’ parents were alive during survey. Bonferroni correction applied to P values, and adjusted significance threshold for tests is 0.05/12=0.004.

‡ Data were available for 42 935 participants.

### Association of early life rationing exposure with cardiovascular outcomes

Table 2 shows the association between early life exposure to sugar rationing and cardiovascular outcomes. For cardiovascular disease, compared with people never exposed, the hazard ratio in model 3 decreased from 0.89 (95% confidence interval (CI) 0.82 to 0.97) for exposure in utero only to 0.86 (0.79 to 0.95) for exposure in utero plus one year, and further to 0.80 (0.73 to 0.90) for exposure in utero plus one to two years (P for trend<0.001). For exposure in utero plus one to two years, the risk was reduced for myocardial infarction (hazard ratio 0.75, 95% CI 0.63 to 0.90), heart failure (0.74, 0.59 to 0.95), atrial fibrillation (0.76, 0.66 to 0.92), stroke (0.69, 0.53 to 0.89), and cardiovascular disease mortality (0.73, 0.54 to 0.98).

**Table 2:** Associations of sugar rationing with risk of various cardiovascular outcomes

	Not rationed	In utero	In utero + 0-1 year	In utero + 1-2 years	P for trend
**Cardiovascular disease**					
Total cases/total sample size	1980/23 370	877/10 466	1275/14 685	1283/14 912	-
Model 1	Reference	0.90 (0.83 to 0.99)	0.88 (0.80 to 0.97)	0.82 (0.74 to 0.92)	<0.001
Model 2	Reference	0.87 (0.80 to 0.96)	0.86 (0.78 to 0.95)	0.79 (0.72 to 0.89)	<0.001
Model 3	Reference	0.89 (0.82 to 0.97)	0.86 (0.79 to 0.95)	0.80 (0.73 to 0.90)	<0.001
**Myocardial infarction**					
Total cases/total sample size	791/23 370	312/10 466	446/14 685	426/14 912	-
Model 1	Reference	0.84 (0.73 to 0.96)	0.83 (0.71 to 0.96)	0.76 (0.63 to 0.91)	0.004
Model 2	Reference	0.83 (0.72 to 0.96)	0.81 (0.69 to 0.94)	0.74 (0.62 to 0.89)	0.001
Model 3	Reference	0.85 (0.74 to 0.98)	0.82 (0.70 to 0.94)	0.75 (0.63 to 0.90)	0.002
**Heart failure**					
Total cases/total sample size	515/23 370	198/10 466	311/14 685	323/14 912	-
Model 1	Reference	0.78 (0.65 to 0.93)	0.81 (0.67 to 0.97)	0.77 (0.62 to 0.97)	0.04
Model 2	Reference	0.77 (0.63 to 0.92)	0.76 (0.62 to 0.93)	0.75 (0.60 to 0.95)	0.02
Model 3	Reference	0.76 (0.62 to 0.92)	0.75 (0.61 to 0.93)	0.74 (0.59 to 0.95)	0.01
**Atrial fibrillation**					
Total cases/total sample size	1084/23 370	518/10 466	725/14 685	808/14 912	-
Model 1	Reference	0.92 (0.82 to 1.03)	0.82 (0.73 to 0.93)	0.81 (0.70 to 0.94)	0.003
Model 2	Reference	0.88 (0.78 to 0.99)	0.80 (0.71 to 0.91)	0.78 (0.68 to 0.93)	<0.001
Model 3	Reference	0.86 (0.76 to 0.97)	0.79 (0.70 to 0.90)	0.76 (0.66 to 0.92)	<0.001
**Stroke**					
Total cases/total sample size	406/23 370	165/10 466	243/14 685	235/14 912	-
Model 1	Reference	0.82 (0.67 to 0.99)	0.80 (0.65 to 0.99)	0.71 (0.55 to 0.92)	0.01
Model 2	Reference	0.81 (0.67 to 0.99)	0.79 (0.64 to 0.98)	0.70 (0.54 to 0.90)	<0.001
Model 3	Reference	0.80 (0.66 to 0.98)	0.77 (0.62 to 0.97)	0.69 (0.53 to 0.89)	<0.001
**Cardiovascular disease mortality**					
Total cases/total sample size	311/23 370	129/10 466	198/14 685	193/14 912	-
Model 1	Reference	0.84 (0.67 to 1.05)	0.86 (0.68 to 1.09)	0.78 (0.58 to 1.04)	0.11
Model 2	Reference	0.82 (0.65 to 1.03)	0.81 (0.63 to 1.03)	0.74 (0.54 to 0.99)	0.10
Model 3	Reference	0.81 (0.64 to 1.03)	0.80 (0.62 to 1.02)	0.73 (0.54 to 0.98)	0.09

Model 1 and model 2 were Cox proportional hazard models. Model 1 adjusted for age and sex. Model 2 included age, sex, race, birth location, calendar month of birth, real food prices (adjusted for Consumer Price Index), parental disease history (cardiovascular disease, diabetes, hypertension), genetic risk score for each outcome, maternal smoking around birth, whether breastfed as baby, and survey year. Model 3 includes same covariates as model 2 but estimates parametric hazard model based on Gompertz distribution.

**Fig 3:**
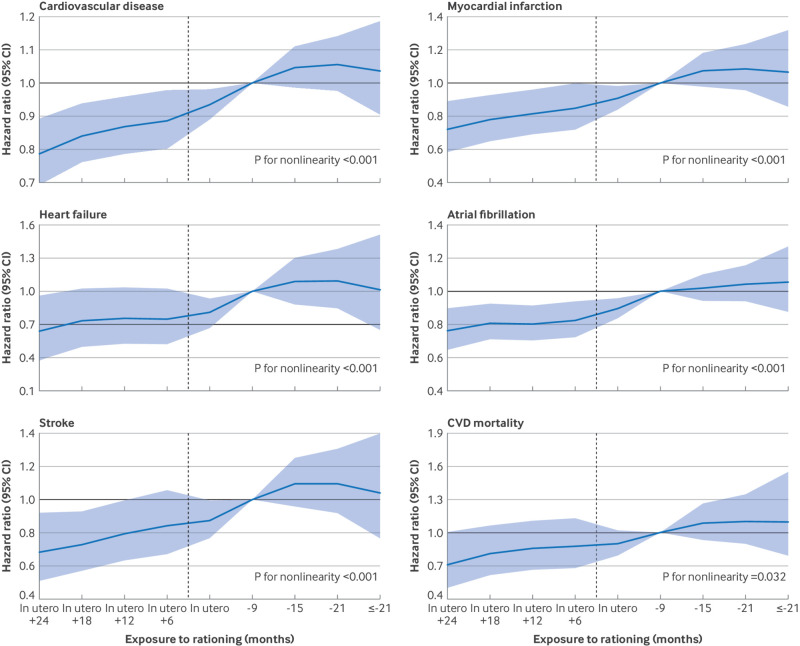
Hazard ratios for different cardiovascular outcomes by various levels of exposure to sugar rationing. Parametric hazard models based on Gompertz distribution were used. Groupings in figure are consistent with those in fig 1. X axis represents duration of participants’ exposure to sugar rationing, with negative values indicating number of months elapsed since rationing ended at time of birth. Model was adjusted for age, sex, race, birth location, calendar month of birth, real food prices (adjusted for Consumer Price Index), parental disease history (cardiovascular disease (CVD), diabetes, hypertension), genetic risk score for each outcome, maternal smoking around birth, whether breastfed as baby and survey year. Hazard ratio estimates for adults born between January 1955 and April 1956, who were never rationed, were not individually or jointly significantly different from estimate for adults born in reference group of July to December 1954 (at P=0.226, 0.293, 0.544, 0.410, 0.610, and 0.613 for CVD, myocardial infarction, heart failure, atrial fibrillation, stroke, and CVD mortality, respectively). Shaded area represents 95% confidence interval (CI). Vertical dashed line indicates end of sugar rationing

The cubic spline curves show the hazard ratio estimates across specific time intervals, with people born between July and December 1954 set as the reference group (fig 3). From in utero plus 24 months to in utero only, hazard ratio values remained below 1 but gradually approached 1. This trend was consistent across different cardiovascular outcomes (P for nonlinearity<0.05 for all outcomes). By contrast, people who did not experience sugar rationing showed no significant difference in cardiovascular risk compared with the reference group. A comparison of incidence of cardiovascular disease among people born at different time points without sugar rationing showed no significant differences (P>0.05).

**Fig 4:**
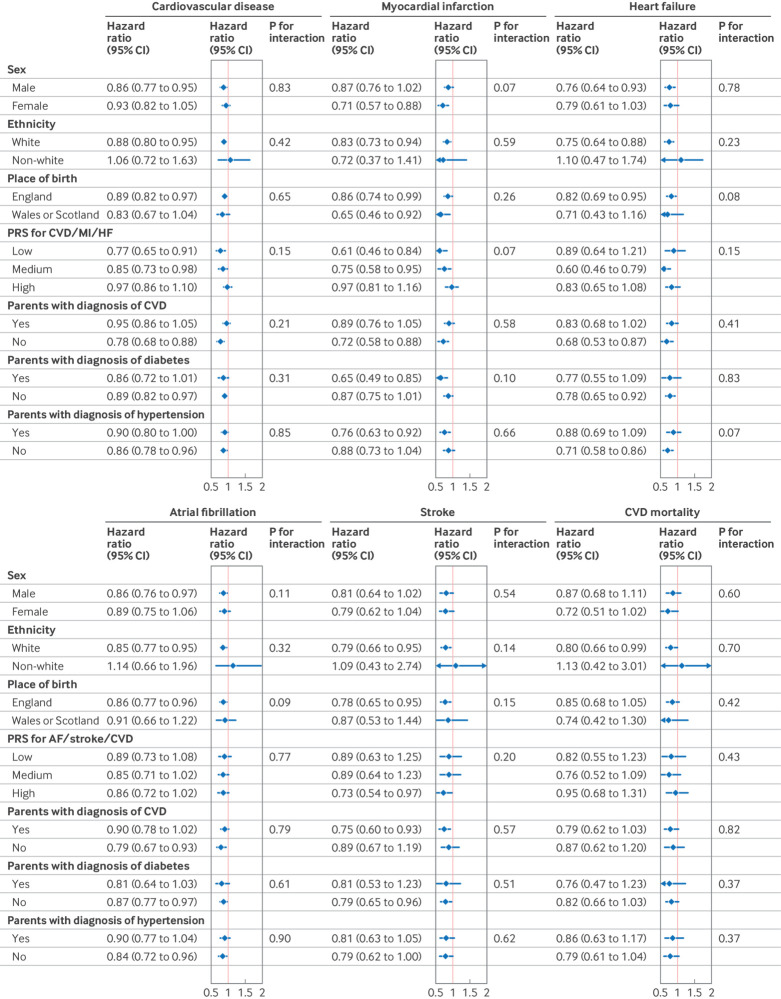
Multivariable stratified analysis of association between exposure to sugar rationing and risk of various cardiovascular outcomes. Parametric hazard models based on Gompertz distribution were used. In polygenic risk score (PRS) stratification, corresponding PRS was used for each outcome; cardiovascular disease (CVD) mortality used CVD specific PRS. Model was adjusted for age, sex, race, birth location, calendar month of birth, real food prices (adjusted for Consumer Price Index), parental disease history (CVD, diabetes, hypertension), genetic risk score for each outcome, maternal smoking around birth, whether breastfed as baby, and survey year. AF=atrial fibrillation; CI=confidence interval; HF=heart failure; MI=myocardial infarction; PRS=polygenic risk score

We did a stratified analysis based on potential risk factors and found that the effect of sugar rationing was not significantly modified by sex, ethnicity, place of birth, polygenic risk score, and parental health (all P for interaction>0.05) (fig 4; supplementary table D).

People exposed to rationing in utero and during early life showed progressively longer delays in the age of onset of cardiovascular outcomes compared with those not exposed to rationing. For cardiovascular disease, the delay in age of onset increased from 0.98 (95% CI 0.66 to 1.3) years for in utero exposure to 2.53 (2.25 to 2.81) years for in utero exposure plus one to two years. We observed similar trends across various outcomes, with the greatest delay in age of onset observed for heart failure (2.96 (95% CI 2.43 to 3.49) years) in people exposed in utero plus one to two years (supplementary table E).

### Sensitivity analysis, placebo tests, and mediation analysis

The main results were consistent after adjustment for later life factors (supplementary table F). After we accounted for competing risks (2697 for cardiovascular disease, 3139 for myocardial infarction, 3037 for heart failure, 2976 for atrial fibrillation, 3181 for stroke, and 2624 for cardiovascular disease mortality), the cumulative incidence curves closely resembled our main analysis (supplementary figure D). People exposed to sugar rationing consistently had lower sub-distribution hazard ratios than their non-rationed counterparts across all cardiovascular outcomes. We found that early life sugar rationing was also associated with lower risk of all cause mortality. Compared with those never exposed, the hazard ratio decreased to 0.77 (95% CI 0.66 to 0.90) for individuals exposed in utero plus one to two years (supplementary figure E).

In placebo analyses using osteoarthritis and cataract as outcomes, we observed no consistent association with early life sugar rationing across exposure durations, and hazard ratios remained centred around 1 (supplementary figure F). We found that incident type 2 diabetes and incident hypertension partially mediated 23.9% and 19.9%, respectively, of the effect of sugar rationing on cardiovascular disease (supplementary figure G). When we incorporated these mediators jointly, they explained 31.1% of the effect, whereas birth weight contributed only 2.2%.

### Contemporaneous validation and control cohorts

The baseline characteristics of the UK Biobank (internal control), HRS (external control), and ELSA (external validation) cohorts are presented in supplementary tables G-I. After matching, the gender and racial proportions of the ELSA and HRS cohorts were similar to those of the main cohort. Supplementary figure H and table J show hazard ratios for various cardiovascular outcomes by date of birth among the UK Biobank control group. Across different birth periods, the hazard ratios remain close to those of the reference group. In the HRS, birth period had no significant association with heart problem (hazard ratio 1.02, 95% CI 0.82 to 1.21; P=0.72) (supplementary figure I). In ELSA, the rationed versus not rationed hazard ratio for heart problem in the all adjusted model was 0.81 (0.64 to 0.98; P=0.04) (supplementary figure I).

### Associations between rationing exposure and MRI indices

Overall, the density curves largely overlap, indicating only minor differences in the distribution of these cardiac indices by rationing status (supplementary figure J). Table 3 shows the associations between exposure to rationing and cardiac MRI indices in the UK Biobank imaging study. After full adjustment, participants who experienced rationing had a small but significant increase in left ventricular stroke volume index (0.73 (95% CI 0.05 to 1.41) mL/m^2^) and a higher left ventricular ejection fraction (0.84%, 0.40% to 1.28%) compared with those not rationed. Participants exposed to early life sugar rationing had lower odds of having low left ventricular ejection fraction (<50%) in adulthood than those not exposed to rationing (adjusted odds ratio 0.81, 95% CI 0.69 to 0.95; P=0.009) (supplementary table K). Differences in left ventricular mass index, left ventricular end diastolic volume index, and left ventricular mass-to-volume ratio were modest and not statistically significant.

**Table 3:** Associations between exposure to sugar rationing and cardiac magnetic resonance imaging indices in UK Biobank imaging study. Values are means (standard deviations) unless stated otherwise

Indicator	Rationed	Not rationed	Model 1			Model 2	
			Rationed *v* not rationed—coefficient (95% CI)	P value		Rationed *v* not rationed—coefficient (95% CI)	P value
LVSVI, mL/m^2^ (n=6710)	46.7 (11.4)	46.1 (11.3)	0.75 (0.07 to 1.43)	0.03		0.73 (0.05 to 1.41)	0.03
LVMI, g/m^2^ (n=801)	45.2 (12.9)	44.1 (12.2)	−0.88 (−2.71 to 0.94)	0.34		−1.04 (−2.89 to 0.77)	0.29
LVEDVI, mL/m^2^ (n=6661)	77.4 (18.2)	77.7 (17.8)	−0.09 (−1.09 to 0.91)	0.85		−0.12 (−1.12 to 0.88)	0.82
LVMVR, g/mL (n=734)	0.57 (0.13)	0.56 (0.11)	−0.012 (−0.037 to 0.012)	0.32		−0.014 (−0.038 to 0.010)	0.26
LVEF, % (n=6717)	54.9 (6.8)	54.6 (6.2)	0.82 (0.38 to 1.26)	<0.001		0.84 (0.40 to 1.28)	<0.001

Link between rationing exposure and cardiac measurements was estimated using ordinary least squares. Model 1 adjusted for age and sex. Model 2 included age, sex, race, birth location, calendar month of birth, real food prices (adjusted for Consumer Price Index), parental disease history (cardiovascular disease, diabetes, hypertension), maternal smoking around birth, whether breastfed as baby, and survey year.

CI=confidence interval; LVEDVI=left ventricular end diastolic volume index; LVEF=left ventricular ejection fraction; LVMI=left ventricular mass index; LVMVR=left ventricular mass-to-volume ratio; LVSVI=left ventricular stroke volume index.

## Discussion

### Principal findings

Our study, leveraging quasi-experimental variation in availability of sugar, found that early life exposure to sugar rationing was associated with lower risks of multiple cardiovascular outcomes, with longer durations of exposure conferring progressively greater protection. Specifically, compared with people who were never exposed, those exposed to rationing in utero plus one to two years experienced a 20% reduction in risk of cardiovascular disease, as well as reduced risks of myocardial infarction (25%), heart failure (26%), atrial fibrillation (24%), stroke (31%), and cardiovascular disease mortality (27%).

This graded association was further reflected in a delayed onset of disease; for instance, participants exposed to sugar rationing in utero plus one to two years developed cardiovascular disease approximately 2.53 years later than their non-exposed counterparts. Hazard ratios among contemporaneous control groups who never experienced rationing remained close to 1, and placebo outcomes (osteoarthritis and cataract) were unaffected by exposure to rationing, thus further supporting the robustness of our findings. Although differences in cardiac MRI parameters were modest, people who experienced rationing showed a small but significant increase in left ventricular stroke volume index (0.73 mL/m^2^) and left ventricular ejection fraction (0.84%). Together, these results highlight the lasting cardiovascular benefits of constrained sugar exposure during the first 1000 days after conception.

### Comparison with other studies

Building on the foundation established by Gracner and colleagues,^29^ our study extends this work by systematically evaluating the association between early life sugar rationing and a comprehensive range of cardiovascular outcomes, thus extending the “fetal origins of disease” hypothesis into the cardiovascular domain.^44^ ^45^ ^46^ Previous work by van den Berg and colleagues focused on the brief de-rationing of sweet confectionery in 1949, finding modest benefits in educational attainment, body mass index, and dietary preferences.^47^ However, they found no significant associations between short term exposure to the policy and cardiovascular disease or type 2 diabetes. We build directly on this foundation by examining the effect of sustained sugar rationing throughout the full first 1000 days of life, including both prenatal and postnatal periods. Our observations suggest that in utero exposure to sugar rationing contributed meaningfully, although not exclusively, to the observed cardiovascular benefits.

Several previous findings align with our observation. Earlier work by Gertler and Gracner, using ELSA data, showed that early life rationing reduced the prevalence of elevated cholesterol by 7.4% and cardiovascular events by 4.1%, highlighting a close link between early life sugar restriction and long term lipid and cardiovascular health.^21^ Additionally, Gracner and colleagues showed that early life sugar rationing may protect against diabetes and hypertension,^29^ both of which are major risk factors for cardiovascular disease. In non-diabetic populations, higher maternal glucose concentrations were found to be associated with elevated childhood blood pressure and risk for congenital heart disease in offspring (8% higher risk per 10 mg/dL increase in glucose).^22^ ^23^ Other research has linked excessive maternal sugar intake to elevated metabolic dysfunction.^48^ ^49^ Moreover, maternal conditions related to sugar intake are closely relevant to the offspring’s cardiovascular health. For example, children of obese mothers were recorded to have increased carotid intima-media thickness and left ventricular concentric remodelling,^50^ ^51^ suggesting a transgenerational impact of maternal obesity. Furthermore, multiple studies indicate that (pre)gestational diabetes can functionally programme fetal organ systems,^52^ ^53^ increasing cardiovascular alterations. Notably, lower maternal energy intake during pregnancy may heighten a child’s susceptibility to atherogenesis.^54^ In our study, total calorie intake changed by less than 5% and most change in calorie intake was caused by change in sugar intake, allowing for a relatively clean sugar specific perspective. By establishing population based evidence, our study fills the gap in how early life sugar restriction influences long term cardiovascular risks. The observed lasting cardiovascular advantages for sugar restriction in utero and infancy underscore the importance of both maternal nutrition and the broader early life dietary environment in shaping long term cardiovascular risk.

Gracner and colleagues observed that early life sugar rationing reduced risk of diabetes and hypertension by approximately 35% and 20%, respectively^29^—two key risk factors for cardiovascular disease. Our mediation analysis suggested that although type 2 diabetes and hypertension jointly explain a fraction of the sugar rationing-cardiovascular disease association, most of the association between sugar rationing and cardiovascular risk may go beyond the pathways of diabetes and hypertension. Although previous research has found a close link between birth weight and chronic diseases in adulthood,^55^ birth weight seems to play a relatively minor role in the sugar rationing-cardiovascular disease association, supporting the idea that nutritional quality during early development, rather than birth size alone, could be a more critical determinant of long term cardiovascular health. Future research should investigate alternative pathways linking sugar rationing and cardiovascular outcomes.

A meta-analysis indicates that adverse experiences during the first 1000 days of life may lead to adaptive changes in infants’ vascular walls and increase carotid intima-media thickness,^56^ highlighting the nutritional programming of cardiac architecture during critical developmental windows. In our imaging analysis, individuals exposed to early life sugar rationing showed small but meaningful increases in left ventricular stroke volume index and left ventricular ejection fraction compared with those never rationed. These findings align with a longitudinal imaging study highlighting how higher gestational glucose concentrations can be linked to a reduced ejection fraction at age 4.^22^ Moreover, the offspring of women with obesity during pregnancy showed reduced left ventricular strain from fetal life through infancy.^24^ In our study, the observed ventricular functional enhancements support the “thrifty phenotype” hypothesis, whereby early nutritional constraints programme organ systems for optimal performance under resource limited conditions, potentially explaining our participants’ sustained cardiac advantages.^57^ By specifically linking sugar rationing during the first 1000 days after conception to favourable adult cardiac indices, our results suggest the potential of precisely targeted nutritional strategies in early life to enhance heart function.

### Clinical and policy implications

During the rationing period, sugar allowances for everyone, including pregnant women and children, were notably consistent with modern dietary recommendations. Adult sugar intake was limited to under 40 g per day, closely aligning with the WHO guideline to keep free sugars below 10% of total daily energy intake (approximately 50 g for a 2000 kcal diet).^58^ Importantly, during this period, no added sugars were permitted for infants under 2 years old, a restriction that mirrors updated guidelines emphasising the importance of minimising sugar intake for infants under 2.^59^ By inadvertently mirroring these contemporary nutritional principles, our findings transcend the historical context of sugar rationing and show the cardiovascular impact from present day limits advocated by WHO, the US dietary guidelines, and the American Heart Association.^58^ ^59^ ^60^

### Biological plausibility of findings

Biological mechanisms underlying the sugar rationing-cardiovascular disease association can differ by developmental stage. During the in utero phase, maternal insulin and metabolic states may influence fetal development through potential epigenetic modifications, hormonal imbalances, or organ reprogramming.^5^ ^6^ For the stage of breastfeeding, increased maternal consumption of added sugars postpartum has been positively associated with elevated insulin concentrations in human milk, which could affect infancy.^61^ Once infants begin consuming solid foods, they may encounter highly processed products rich in added sugars, thereby shaping taste preferences and metabolic trajectories.^21^ By spanning the entire early life window from pregnancy through infancy, our design did not precisely capture these varying exposures but revealed their collective effect on long term cardiovascular risks. The progressively stronger protective associations we observed as exposure continued from conception to after birth underscore the significance of both fetal and postnatal factors, albeit through distinct biological pathways.

Although the exact mechanisms underlying the protective effects of early life sugar rationing remain incompletely understood, several plausible mechanisms have been proposed. Reduced maternal hyperglycaemia during pregnancy may decrease fetal insulin secretion, preventing adverse adaptations such as cardiomyocyte hypertrophy, vascular stiffness, and altered cardiac remodelling.^62^ Reduced maternal sugar intake may also modulate oxidative stress and inflammation, key factors in fetal vascular development. Elevated glucose concentrations increase reactive oxygen species and activate pro-inflammatory pathways, such as NF-κB (nuclear factor κ light chain enhancer of activated B cells), in the placenta,^63^ impairing endothelial function and vascular reactivity. Constrained sugar exposure during critical windows may reduce concentrations of reactive oxygen species and preserve the bioavailability of nitric oxide.

### Strengths and limitations of study

Our study has several strengths. Firstly, building on the quasi-experimental design established by Gracner and colleagues,^29^ we applied this design to a broad spectrum of cardiovascular outcomes in adulthood and integrated clinical cardiovascular endpoints with detailed cardiac MRI data, enabling the assessment of both subclinical and clinical effects of early life exposure to sugar rationing. Secondly, we did mediation analyses to explore the potential pathways, such as diabetes, hypertension, and birth weight, linking early sugar restriction to later cardiovascular risk. Thirdly, we have comprehensive information on socioeconomic status, lifestyle behaviours, parental health, and genetic data, and we consistently observed the protective effect of sugar rationing across various analytical models and specifications. Fourthly, our large sample size allowed us to separately identify the effects of in utero exposure and in utero plus postnatal exposure, even while using narrow analytical windows. This increases the precision of our results. Finally, we used two external cohorts as the validation and control groups respectively. The results indicate that the association is not a chance finding in a single cohort and that this finding cannot be replicated in countries where sugar rationing has not been implemented, reducing the likelihood that global secular trends explain the results.

Despite its strengths, this study has several limitations. Our study involved cohorts from an earlier era, so caution is warranted when extrapolating to modern populations with different eating habits. Although changes in dietary habits have occurred, basic food items such as sugar always occupy an important position. Hence, such changes are unlikely to overturn our main findings. Concerns exist about the influence of general time trends or improved disease detection over time. Nevertheless, disease risks for non-rationed adults born after December 1954 were similar, with hazard ratios consistently around 1, suggesting that the findings are not driven by these temporal factors. Sweet rationing ended a few months earlier than sugar rationing and may have influenced the results. However, the total consumption of sweets was relatively small (less than one fifth of sugar consumption), and their main ingredient is sugar itself. If this timing discrepancy had any effect, it would likely attenuate the hazard ratios, making our results more conservative rather than inflating them. Regarding potential spurious correlations, we re-estimated the full model using placebo outcomes, such as osteoarthritis and cataract, which showed no significant associations. A common concern in such studies is the presence of unobserved differences between groups during and after the sugar rationing period. To overcome this, we established contemporaneous control groups consisting of individuals born outside the UK, enabling more reliable comparisons.

Although animal studies with mechanistic analysis align with our findings and we have done mediation analyses on multiple pathways, the biological mechanisms linking exposure to outcomes remain largely unknown and warrant further basic research to elucidate. Furthermore, although the UK Biobank provides a large and detailed dataset for studying exposure-outcome relations, it lacks national representativeness,^64^ as participants tend to be wealthier and healthier than the general population. Nevertheless, the consistent recruitment protocols for rationed and non-rationed cohorts mitigate concerns about differential selection bias. Furthermore, we also verified the association between sugar rationing and long term cardiovascular risk in the ELSA cohort, which strengthens the generalisability of our findings. The study is subject to right-censoring and lacks pre-study mortality data. However, given the similarly low mortality rates among rationed and non-rationed adults, this concern is likely minimal.^65^ Early life factors such as birth weight, maternal smoking, and breastfeeding were subject to recall bias and a certain amount of missing data. However, insufficient evidence exists to suggest a strong correlation between these factors and birth period, especially within the relatively narrow study window of our cohort. Furthermore, the distributions of early life factors seem relatively balanced between the rationed and non-rationed groups, with no significant differences, suggesting that they are not very likely to have a substantial impact on our results. However, the results of birth weight in the mediation analysis should be interpreted with caution.

Owing to the unique sugar rationing policy ending in the 1950s and the constraints of that era, detailed individual level food intake data were not available. We recognise that national averages can mask substantial individual variability in sugar consumption. We used multiple data sources and did subgroup analyses by sex, race, residence, genetics, and parental health, finding consistently similar trends in different population groups. After adjusting for many covariates, we observed a unified trend between timing of exposure to sugar rationing and outcomes, suggesting a policy level rather than individual difference driven effect. Moreover, socioeconomic differences in sugar intake variation were minimal across social classes (A, B, C, D) (supplementary figure A), indicating broad and relatively even coverage by this policy. Future randomised controlled trials with prospectively collected detailed, individual level dietary data are warranted to validate our findings. We also note that alcohol consumption was measured less precisely, as the questionnaire recorded frequency categories rather than absolute amounts. Although the questionnaire is widely used and validated,^66^ ^67^ ^68^ potential reporting biases and the imprecision should be considered when interpreting results. Potential confounding from concurrent changes, such as the de-rationing of other food categories,^69^ shifts in total calorie intake, and variations in purchasing power, should also be considered. To overcome these problems, we adjusted for intake of fat—the food category showing the largest change—and incorporated a Consumer Price Index adjusted real food price index; our results remained robust under these conditions. Furthermore, total calorie intake changed by less than 5%, with most of that difference stemming from sugar and fat. Consequently, following our comprehensive adjustment strategy, this analysis can still be considered a relatively clean natural experiment for evaluating sugar specific effects.

Lastly, results for the final exposure group (≤21 months) in figure 2 and for non-white participants should be interpreted with caution owing to smaller sample sizes and limited event counts, which may lead to instability in risk estimates. Nevertheless, our main analysis using broader exposure categorisations (for example, in utero only, in utero plus one to two years) mitigated this instability and reinforces the robustness of the overall observed risk pattern.

### Conclusions

Our findings showed that constrained sugar exposure in utero and during infancy, particularly with longer durations of exposure, provided progressively greater protection against the risks of multiple cardiovascular outcomes and delayed disease onset. Our results underscore the cardiac benefit of early life policies focused on sugar rationing during the first 1000 days after conception. Our findings provide implications for future randomised controlled trials targeting more refined interventions and mechanistic studies in each developmental phase. Further studies should investigate individual level dietary exposures and consider the interplay between genetic, environmental, and lifestyle factors to develop more personalised prevention strategies.

## What is already known on this topic

The first 1000 days after conception are a critical window when nutrition shapes lifelong cardiometabolic risk
Many infants and toddlers consume excess added sugars via maternal diet, formula, and early solids
Evidence in humans on whether early life sugar restriction affects cardiovascular risk in adulthood has been limited and indirect

## What this study adds

Early life sugar restriction was associated with lower risks of myocardial infarction, heart failure, atrial fibrillation, stroke, and cardiovascular mortality
Modest improvements were seen in cardiac imaging markers such as higher left ventricular stroke volume index and ejection fraction
Mediation analysis suggested that diabetes and hypertension jointly explained ~30% of the association, with minimal role of birth weight

## Data Availability

Data are available in a public, open access repository: https://github.com/yangleipzig/sugar-rationing-CVD-analysis-code.

## Web extra

Extra material supplied by authors

Web appendix: Supplementary materials

## Notes

Contributors: CY and GYHL are joint senior authors. JZ, CY, and GL designed the study. JZ, ZZ, and JH collected the data. JZ, ZZ, QT, HW, PQ, WH, and JS contributed to the analysis or interpretation of the data. JZ, ZZ, and QY prepared the first draft of the manuscript. All authors contributed to the interpretation of the results and to critical revision of the manuscript for important intellectual content and approved the final version of the manuscript. CY is the guarantor. The corresponding author attests that all listed authors meet authorship criteria and that no others meeting the criteria have been omitted.

Transparency: The lead author (the manuscript’s guarantor) affirms that the manuscript is an honest, accurate, and transparent account of the study being reported; that no important aspects of the study have been omitted; and that any discrepancies from the study as planned (and, if relevant, registered) have been explained.

Dissemination to participants and related patient and public communities: This study used data from the UK Biobank, which has a broad dissemination strategy in place for participants and the public. Beyond this, the research findings will be disseminated to the wider community through press releases, social media platforms, presentations at international forums, and reports to relevant government agencies and academic societies. Additionally, we will present this study at some well known academic conferences and will continue to engage with journalists, particularly when discussing the cardiovascular health effects of early life sugar restriction.

Provenance and peer review: Not commissioned; externally peer reviewed.
