# CORRECTION: Exposure to sugar rationing in first 1000 days after conception and long term cardiovascular outcomes: natural experiment study

**DOI:** 10.1136/bmj.s119

**Published:** 2026-01-20

In this paper by Zheng and colleagues (*BMJ* 2025;391:e083890, doi:10.1136/bmj-2024-083890, published 22 October 2025), the data sharing statement was corrected after publication to add a direct link to the dataset in the Github open access repository.

