# Supplementary material for: Exposure to sugar rationing in first 1000 days after conception and long term cardiovascular outcomes: natural experiment study
Source: BMJ. 2025 Oct 22;391:e083890. doi: 10.1136/bmj-2024-083890 (PMC12542096; doi:10.1136/bmj-2024-083890)
Supplement: Supplementary file 1 — Web appendix: Supplementary materials [file zhej083890.ww1.pdf]

Supplemental Content

eMethods. 2
Supplementary Table A. The numbers (percentages) of participants with missing covariates 7
Supplementary Table B. Assessing the best fitted distribution using the Akaike and Bayesian Information Criterion. 8
Supplementary Table C. Comparison of later-life socioeconomic, lifestyle, and health-related variables between rationed and non-rationed participants 9
Supplementary Table D. Multivariable stratified analysis of the association between rationing exposure and risk of various cardiovascular outcomes. 10
Supplementary Table E. The effect of rationing on age of disease onset by duration of exposure to rationing. 14
Supplementary Table F. Hazard ratios for cardiovascular outcomes across early-life sugar rationing exposure categories under the model including later-life lifestyle and health factors. 15
Supplementary Table G. Baseline characteristics of individuals born outside the UK who have not experienced sugar rationing, compared with participants born in the UK. 16
Supplementary Table H. Baseline characteristics of participants in external validation cohort (ELSA). 17
Supplementary Table I. Baseline data for the contemporaneous control group (HRS), including individuals without exposure to sugar rationing. 18
Supplementary Table J. Cardiovascular outcomes among non-UK born participants 19
Supplementary Table K. Odds of low left ventricular ejection fraction (<50%) by sugar rationing exposure group 20
Supplementary Figure B. Directed Acyclic Graph.. 22
Supplementary Figure C. Comparative density distributions of PRS (CVD, MI, HF, AF, stroke) between rationed and not rationed groups. 23
Supplementary Figure E. Association between sugar rationing exposure and all-cause mortality. 25
Supplementary Figure F. Hazard ratios for placebo outcomes (osteoarthritis [n=9,815] and cataract [n=4,187]) by various levels of rationing exposure. 26
Supplementary Figure G. The standard mediation analysis investigating the proportion mediated by diabetes, hypertension, and birth weight in the relationship between sugar rationing and cardiovascular disease. 27
Supplementary Figure I. Association between sugar rationing exposure and cumulative incidence of heart problem in ELSA and HRS. 29
Supplementary Figure J. Comparative Density Distributions of Cardiac Metrics (LVSVI, LVMI, LVEDVI, LVMVR, LVEF) Between Rationed and Not Rationed Groups. 30

eMethods

Details on statistical analysis

As previously implemented by Gracner et al.,[1] we employed an event study framework to assess the long-term cardiovascular impact of early-life exposure to sugar rationing. Our parametric hazard model followed a similar specification, based on the Gompertz distribution, to estimate hazard ratios (HRs) comparing various exposure cohorts.

We specified the model as follows:

$$h(t)_i = \exp\left(\sum_{k=-3,k\neq 0}^{5} \beta_k \cdot (\text{Birthgroup}_k = 1) + \theta' X_i + \mu_m + \delta_t\right) \cdot \exp(\gamma t)$$

Here, $h(t)$ is the hazard rate at time t, and γ is the shape parameter of the Gompertz function. The variable Birthgroup_k indicates the cohort to which an individual belongs, based on their month and year of birth relative to the end of sugar rationing. The reference group ($k = 0$) consists of individuals born between July and December 1954, who were not exposed to rationing.

Exposure was stratified into multiple categories:

- $k = 1$: in utero exposure (Oct 1953–Jun 1954);
- $k = 2$: in utero and up to age 6 months (Apr–Sep 1953);
- $k = 3$: in utero and up to age 12 months (Oct 1952–Mar 1953);
- $k = 4$: in utero and up to age 18 months (Apr–Sep 1952);
- $k = 5$: in utero and up to age 24 months (Oct 1951–Mar 1952);
- $k = -1, -2, -3$: individuals born after the official end of rationing (Jan 1955–Mar 1956), grouped into three 6-month bins.

We interpreted β_k coefficients as relative hazard ratios for each exposure group, compared to the reference cohort ($k = 0$). All models adjusted for covariates $\theta' X_i$, including age, sex, race, region of birth (England, Wales, Scotland), geographic coordinates, calendar birth month (fixed effects), household income, CPI-adjusted food prices, parental medical history, parental survival status, maternal smoking during pregnancy, breastfeeding status, polygenic risk score, and survey year (δ_t). Calendar birth month (μ_m) adjusted for seasonality.

To examine life stage-specific effects while improving statistical power, we also collapsed exposure groups into four categories using a simplified model:

$$h(t)_i = \exp\left(\sum_{k=1}^{3} \beta_k \cdot \text{Birthgroup}_k + \theta' X_i + \mu_m + \delta_t\right) \cdot \exp(\gamma t)$$

In this formulation, $k = 0$ again denotes the never-rationed cohort (born after July 1954). Exposure groups included: in utero only ($k = 1$), in utero plus up to 1 year ($k = 2$), and in utero plus 1–2 years ($k = 3$). These categories are reflected in Table 2.

We included non-rationed cohorts to evaluate potential secular trends and to verify the consistency of hazard rates in populations not affected by early-life sugar restrictions. This approach helps address the possibility that improvements in diagnostics or treatment might otherwise confound risk comparisons across birth cohorts.

Details for post-treatment variables

Average social fat intake was calculated using the average fat intake recorded in the two quarters before and after birth (derived from the NSF data). Physical activity was collected via an electronic questionnaire and defined by Total Metabolic Equivalent Task (MET) minutes per week for all activities, including walking, moderate, and vigorous physical activities. The MET score was derived based on the guidelines of the International Physical Activity Questionnaire (IPAQ). Body mass index (BMI) was calculated by dividing a person's weight in kilograms by the square of their height in meters (kg/m^2). The Townsend deprivation index is a measure of social and economic disadvantage based on factors like employment, car ownership, housing, and household overcrowding.[2]

Genotyping and quality control

UK Biobank samples (version 3; March 2018) were genotyped for > 800,000 SNPs using either the Affymetrix UK BiLEVE Axiom array or the Affymetrix UK Biobank Axiom array. Imputation via IMPUTE2 was carried out centrally by UK Biobank researchers using the merged 1000 Genomes Project panel and UK 10K panel.[3] After imputation, variant-level quality control (QC) was performed by filtering SNPs on two criteria: (1) minor allele frequency < 0.01 and (2) imputation quality score < 0.3. A total of 9,505,768 imputed autosomal SNPs passed the QC criteria. We used multiple imputation to fill in the missing data or data excluded during quality control. The polygenic risk scores for CVD, MI, HF, AF and stroke were formulated using an additive approach, as previously detailed. The count of alleles (0, 1, or 2) per individual was summed after being multiplied by the effect size pertaining to the association between the specific SNP and the respective disease. PRS was calculated using the formula: $PRS=\beta_1\times SNP_1+\beta_2\times SNP_2+\ldots+\beta_n \times SNP_n$. SNP_i represents the risk allele number of each SNP.[4] A higher PRS suggests a greater genetic susceptibility to the disease. Participants with PRS scores in the lowest quintile were classified as "low", those in the second to fourth quintiles as "medium", and those in the highest quintile as "high". These effect sizes were derived from established genome wide association studies that had been previously published. [5-8] Given that certain studies identified multiple related variants within the same genomic region, independent SNPs were chosen based on the highest reported p value. This selection was conducted using the linkage disequilibrium clumping method (at $R^2 < 0.01$) available in PLINK version 1.9 (https://www.cog-genomics. org/plink2).

Cardiac magnetic resonance imaging protocol and analysis

Recruitment for the imaging subsection of the UKB was conducted through a randomized invitation strategy.[9] Detailed previously, the standardized protocol for Cardiac Magnetic Resonance (CMR) acquisition employs 1.5-Tesla Siemens Healthineers scanners (Erlangen, Germany).[10] Short-axis cine stacks were analyzed through automated segmentation utilizing a deep learning neural network, which has achieved parity in performance with human experts and has been specifically optimized for UKB image datasets.[11] Subsequent to segmentation, these images were subjected to post-processing to quantify biventricular volumes at end-systole and end-diastole, as well as stroke volumes, adhering to standardized protocols.[12] Although cardiac MRI is highly accurate and reproducible in measuring absolute cardiac structure sizes,

these measurements are significantly influenced by body size.[13] To evaluate relative differences independent of body size, measurements are commonly adjusted by indexing to body surface area (BSA), which is determined using the Mosteller formula.[14] Raw values of cardiac phenotypes are then indexed by division by BSA. Of the 12 cardiac phenotypes quantified, following conventions in cardiac MRI literature,[15] the following were indexed to BSA: left ventricular stroke volume index (LVSVI), left ventricular mass index (LVMI) and left ventricular end-diastolic volume index (LVEDVI).

Mediation analysis

We applied a four-step mediation procedure (Kenny and Baron 4 step analysis)[16] to investigate whether diabetes, hypertension, and birth weight served as mediators in the relationship between sugar rationing (exposure, X) and cardiovascular disease (outcome, Y). First, we examined the Total Effect by regressing CVD on sugar rationing alone, establishing the overall relationship. Second, we tested the Path X → M by verifying that sugar rationing significantly affected each proposed mediator (diabetes, hypertension, and birth weight). Third, we evaluated Path X + M → Y to determine whether each mediator influenced CVD while accounting for sugar rationing. Finally, we quantified how much of the total effect was indirectly mediated by these variables.

To perform these analyses, we collected data on 4,086 cases of diabetes and 19,443 cases of hypertension that occurred before the onset of 6,523 CVD cases, of which 5,415 arose during follow-up. We identified cases using self-reported questionnaires (data fields 2443 and 1065) and diagnostic records from primary care and hospital databases (data field 130708 and 131286 or 131294). Through hospital linkages, diseases were classified based on International Classification of Diseases, 10th Revision (ICD-10) codes, with E11 used for type 2 diabetes (T2DM) and I10/I15 for hypertension. We then fit linear regression models (adjusting covariates as specified in Model 2) and used the mediate() function from the mediation package in R. This function employs 1,000 bootstrap simulations to generate robust estimates of direct and indirect effects. Additionally, we used structural equation modeling (SEM) through the lavaan package to integrate diabetes and hypertension as parallel mediators in a single model, thereby estimating the proportion of the sugar rationing effect simultaneously attributed to both conditions.

External validation cohort and control cohort

The English Longitudinal Study of Ageing (ELSA): ELSA is an ongoing, multidisciplinary panel study of a nationally representative cohort of adults living in England.[17] ELSA was established in 2002 as a sister study to the Health and Retirement Study (HRS) in the United States, and it collects comprehensive data on economic circumstances, social and psychological wellbeing, cognitive function, health status, biomarkers, and genetics. The original sample included 11,391 participants, with refreshment samples of adults aged 50 and over added in subsequent waves to maintain representativeness of the aging population. ELSA data are collected every two years through computer-assisted personal interviews and self-completion questionnaires, with additional nurse visits for biomarker collection

starting in Wave 2 (2004–2005) and repeated every four years. For this analysis, we included data from Waves 1 to 9 (2002–2018). ELSA is harmonized with other international aging studies and is linked to financial and health registry records. All participants provided informed consent, and the study was approved by the London Multi-Centre Research Ethics Committee. Further details are available at https://www.elsa-project.ac.uk/data-and-documentation.

Sample selection for the ELSA: We identified 2,157 ELSA participants born between October 1951 and March 1956. After excluding 399 participants (26 born outside the UK, 8 adopted, 58 with missing covariate data, and 307 with prevalent heart problems), 1,758 heart problem-free individuals remained. Following frequency matching with the main UK cohort by sex and race, 1,694 participants were included in the final analysis as an external validation cohort (935 rationed, 759 not rationed).

HRS: HRS is a nationally representative, longitudinal panel study of adults aged 50 years and older in the United States.[18] The HRS began in 1992 and conducts biennial follow-up interviews, either in person or by telephone. The original HRS sample has been expanded through the addition of new birth cohorts, and in 1998, the study undertook a major merging of its early cohorts. As a result, the total HRS core sample in 1998 was 21,384 individuals. For this study, we used data from Waves 4 to 12 (1998–2014), with 1998 (Wave 4) serving as the baseline. The study collects detailed longitudinal information on demographic characteristics, health status, cognitive function, family and social networks, economic variables, and biological samples. All participants provided informed consent, and the study protocol was approved by the University of Michigan Institutional Review Board. Additional information is available at https://hrs.isr.umich.edu/documentation/survey-design.

Sample selection for HRS: We identified 3,464 HRS participants born between October 1951 and March 1956. After excluding 632 participants (516 with prevalent heart problems, 102 with missing covariate data, and 14 adopted), 2,832 heart problem-free individuals remained. After frequency matching with the main UK cohort by sex and race, 1,763 participants were included in the final analysis as external controls (847 rationed, 916 not rationed).

For both cohorts, the follow-up period for all participants began at recruitment and ended at the time of outcome diagnosis, death, loss to follow-up, or the study's end, whichever occurred first.

Variables in ELSA and HRS: In the ELSA study, total household wealth was calculated as the sum of net values from primary residence, business assets, non-housing financial wealth, secondary residence, and other physical assets, constructed at the benefit unit level (couples or single individuals with dependents). For non-response cases ("don't know" or refusal), unfolding bracket questions with consistent wording, amount ranges, and entry points were employed across waves, with a confirmation check for property values exceeding £500,000 in Waves 1-2 that was discontinued after Wave 2. In the HRS, total wealth is calculated as the sum of all asset components, including housing, real estate, vehicles, businesses, financial assets, and retirement accounts, minus all debts such as mortgages, loans, and other liabilities. Smoking status was assessed in both datasets using two standard questions: “Have

you ever smoked cigarettes?" and "Do you currently smoke cigarettes at all nowadays?" Alcohol consumption in ELSA referred to the past 12 months (collected via self-completion questionnaire from Wave 2 onward), while HRS measured consumption over the past 3 months. Disease diagnoses were based on self-reported physician diagnosis at each wave. For estimation, we employed Cox proportional hazards models in both datasets to evaluate the association between early-life rationing exposure and risk of heart problems. In this analysis, we used a simplified adjustment model. Model 1 adjusted for age, sex, and race. Model 2 further included education and marital status. Model 3 additionally controlled for survey year to account for period effects.

Multiple imputation

We utilized the 'mcar_test' function from the 'naniar' R package to assess the Missing at Random assumption. With a p-value > 0.05, the result accepts the Null hypothesis, suggesting that the missingness is completely at random. Additionally, the missing data patterns were less than 5%, indicating that missingness is assumed not to influence the analysis.

Upon examination, no significantly unbalanced variables were identified. Given that the variable types of the missing values in our study are mixed, and considering that 'mice_pmm' (Predictive mean matching) is a less restrictive method compared to Bayesian linear regression, Logistic regression, Polytomous logistic regression, and Proportional odds model, we opted to use the 'mice_pmm' package for imputing missing values.[19] We set the caliper at 5%. We assessed the Fraction of Missing Information (FMI) for Maternal smoking around birth and Breastfeeding, which was low, indicating that missing data had minimal impact on model uncertainty. The Relative Increase in Variance (RVI) was also low, suggesting that multiple imputation did not inflate variability across the imputed datasets.

This approach aligns with common practices in UK Biobank-based studies. The method we employed is consistent with the default recommendation from the 'mice' package. In total, we imputed twenty datasets, incorporating all variables utilized in the analysis. Notably, we observed no significant deviations between the imputed and observed values.

Supplementary Table A. The numbers (percentages) of participants with missing covariates

**Covariates**	**n**	**%**
Breastfed as a baby	10560	16.6
Maternal smoking around birth	7853	12.4
Household income	5823	9.18
Parental health information	3235	5.10
Genotyping	2067	3.26
Physical activity	330	0.52
Body mass index	266	0.42
Ethnicity	196	0.31
Smoking status	177	0.28
Townsend Deprivation Index	44	0.07

Supplementary Table B. Assessing the best fitted distribution using the Akaike and Bayesian Information Criterion.

	**Gompertz**	**Weibull**	**Logistic**	**Log normal**	**Exponential**
**CVD**					
AIC	28893.23	28920.18	30677.12	29270.14	29352.18
BIC	28900.62	28932.37	30688.92	29282.33	29355.27
**MI**					
AIC	50742.53	50790.44	51369.56	50874.52	51051.57
BIC	50747.46	50798.25	51377.25	50882.32	51050.32
**HF**					
AIC	35252.31	35449.89	35588.08	35554.19	35862.63
BIC	35263.37	35460.79	35598.95	35565.06	35864.42
**AF**					
AIC	77470.66	77633.03	78337.15	77842.15	78118.73
BIC	77463.63	77635.46	78339.43	77844.53	78112.04
**Stroke**					
AIC	28202.48	28320.14	28531.13	28392.44	28451.32
BIC	28205.47	28332.47	28543.42	28404.76	28454.61
**CVD mortality**					
AIC	21906.73	22667.7	22742.29	22724.64	22933.50
BIC	21917.03	22681.16	22755.73	22738.09	22937.89

Based on this criterion we proceeded with Gompertz distribution as our preferred one. AIC = Akaike information criterion; BIC = Bayesian information criterion. CVD = cardiovascular disease; MI = myocardial infarction; HF = heart failure; AF = atrial fibrillation.

Supplementary Table C. Comparison of later-life socioeconomic, lifestyle, and health-related variables between rationed and non-rationed participants

	Total	Rationed	Not Rationed	Difference (percentage points)	P value
**No. of participants**	63433	40063	23370		
**Education, n (%)**					
Below A levels	26127 (41.2)	16647 (41.6)	9480 (40.6)	1.0	<0.001
A levels (high school)	19945 (31.4)	12287 (30.7)	7658 (32.8)	-2.1	
College/university	7818 (12.3)	4960 (12.4)	2858 (12.2)	0.2	
Professional/other	9543 (15.0)	6169 (15.4)	3374 (14.4)	1.0	
**Household income, n (%),** £					
<18 000	14886 (23.5)	9772 (24.4)	5114 (21.9)	2.5	<0.001
18 000-30 999	11785 (18.6)	7666 (19.1)	4119 (17.6)	1.5	
31 000-51 999	16792 (26.5)	10551 (26.3)	6241 (26.7)	-0.4	
52 000-100 000	15473 (24.4)	9394 (23.4)	6079 (26.0)	-2.6	
>100 000	4497 (7.1)	2680 (6.7)	1817 (7.8)	-1.1	
**Townsend deprivation index, mean (SD)**	-1.5 (3.0)	-1.5 (3.0)	-1.4 (3.0)	-0.1	<0.001
**Smoking status, %**					
Never	35771 (56.4)	22406 (55.9)	13365 (57.2)	-1.3	<0.001
Former	20631 (32.5)	13381 (33.4)	7250 (31.0)	2.4	
Current	7031 (11.1)	4276 (10.7)	2755 (11.8)	-1.1	
**Alcohol consumption frequency, n (%), times/wk**					
< 3	34134 (53.8)	21464 (53.6)	12670 (54.2)	-0.6	0.121
>=3	29299 (46.2)	18599 (46.4)	10700 (45.8)	0.6	
**BMI, mean (SD), kg/m^2**	27.5 (5.0)	27.5 (5.0)	27.4 (5.0)	0.1	0.334
**Summed MET minutes per week for all activity, median (IQR)**	1800 (798-3519)	1806 (795-3532)	1794 (807-3492)	12.1	0.797
**Personal medical condition, n (%)**					
Hypertension	35315 (55.7)	22627 (56.5)	12688 (54.3)	2.2	<0.001
High cholesterol	11278 (17.8)	7388 (18.4)	3890 (16.6)	1.8	<0.001
Diabetes	3288 (5.2)	2127 (5.3)	1161 (5.0)	0.3	0.0641
Digestive disease	513 (0.8)	337 (0.8)	176 (0.8)	0.001	0.251
Kidney disease	1642 (2.6)	1086 (2.7)	556 (2.4)	0.3	0.012
Liver disease	2176 (3.4)	1409 (3.5)	767 (3.3)	0.2	0.122

Variables listed in this table may be influenced by early-life sugar rationing exposure and are presented for descriptive purposes only. They are not used to assess baseline comparability between exposure groups and are excluded from the main model specifications to avoid post-treatment bias. SD = standard deviation; IQR = interquartile range; BMI = body mass index; MET = metabolic equivalent of task.

Supplementary Table D. Multivariable stratified analysis of the association between rationing exposure and risk of various cardiovascular outcomes.

	Event (n)/Rationed participants (N)	Event (n)/Not rationed participants (N)	Rationed vs not rationed--Hazard ratio (95% CI) for model 1	Rationed vs not rationed--Hazard ratio (95% CI) for model 2	Rationed vs not rationed--Hazard ratio (95% CI) for model 3	P for interaction
**CVD**						
**Sex**						
Male	2107/17286	1232/10051	0.87 (0.78-0.96)	0.86 (0.76-0.95)	0.86 (0.77-0.95)	0.834
Female	1328/22777	748/13319	0.94 (0.83-1.06)	0.92 (0.81-1.04)	0.93 (0.82-1.05)	
**Ethnicity**						
White	3297/38612	1897/22417	0.89 (0.81-0.96)	0.87 (0.79-0.94)	0.88 (0.80-0.95)	0.416
Non-white	138/1451	83/953	1.12 (0.76-1.67)	1.09 (0.73-1.64)	1.06 (0.72-1.63)	
**Place of birth**						
England	3003/34563	1696/20018	0.90 (0.83-0.98)	0.88 (0.81-0.96)	0.89 (0.82-0.97)	0.654
Wales or Scotland	432/5500	284/3352	0.86 (0.69-1.07)	0.82 (0.66-1.04)	0.83 (0.67-1.04)	
**PRS for CVD**						
Low	784/12839	441/7443	0.79 (0.68-0.93)	0.77 (0.65-0.90)	0.77 (0.65-0.91)	0.149
Medium	1032/13193	616/7704	0.86 (0.74-0.99)	0.84 (0.72-0.97)	0.85 (0.73-0.98)	
High	1500/12779	849/7503	0.98 (0.67-1.11)	0.96 (0.85-1.09)	0.97 (0.86-1.10)	
**Parents diagnosed with CVD**						
Yes	2257/23425	1227/13209	0.97 (0.88-1.06)	0.95 (0.85-1.05)	0.95 (0.86-1.05)	0.212
No	1178/16638	753/10161	0.79 (0.69-0.89)	0.77 (0.67-0.88)	0.78 (0.68-0.88)	
**Parents diagnosed with diabetes**						
Yes	686/7246	438/4491	0.87 (0.73-1.02)	0.85 (0.71-1.00)	0.86 (0.72-1.01)	0.306
No	2749/32817	1542/18879	0.90 (0.83-0.98)	0.88 (0.81-0.96)	0.89 (0.82-0.97)	
**Parents diagnosed with hypertension**						
Yes	1594/18077	958/11220	0.91 (0.80-1.01)	0.89 (0.79-0.99)	0.90 (0.80-1.00)	0.846
No	1841/21986	1022/12150	0.87 (0.79-0.97)	0.85 (0.77-0.95)	0.86 (0.78-0.96)	
**MI**						
**Sex**						
Male	844/17286	539/10051	0.88 (0.78-1.04)	0.86 (0.75-1.01)	0.87 (0.76-1.02)	0.073
Female	340/22777	252/13319	0.73 (0.59-0.90)	0.70 (0.58-0.88)	0.71 (0.57-0.88)	
**Ethnicity**						
White	1146/38612	757/22417	0.84 (0.74-0.95)	0.83 (0.72-0.94)	0.83 (0.73-0.94)	0.591
Non-white	38/1451	34/953	0.75 (0.40-1.45)	0.70 (0.35-1.39)	0.72 (0.37-1.41)	
**Place of birth**						
England	1036/34563	665/20018	0.88 (0.75-1.01)	0.86 (0.74-0.99)	0.86 (0.74-0.99)	0.260
Wales or Scotland	148/5500	126/3352	0.69 (0.49-0.96)	0.64 (0.45-0.91)	0.65 (0.46-0.92)	
**PRS for MI**						
Low	196/12841	148/7441	0.63 (0.48-0.85)	0.60 (0.44-0.83)	0.61 (0.46-0.84)	0.066
Medium	344/13194	246/7705	0.77 (0.60-0.97)	0.74 (0.57-0.94)	0.75 (0.58-0.95)	
High	602/12776	364/7504	0.98 (0.82-1.18)	0.96 (0.80-1.15)	0.97 (0.81-1.16)	
**Parents diagnosed with CVD**						
Yes	767/23425	485/13209	0.90 (0.77-1.06)	0.88 (0.75-1.05)	0.89 (0.76-1.05)	0.580
No	417/16638	306/10161	0.73 (0.59-0.89)	0.71 (0.57-0.87)	0.72 (0.58-0.88)	
**Parents diagnosed with**						

**diabetes**						
Yes	239/7246	192/4491	0.69 (0.53-0.88)	0.65 (0.49-0.85)	0.65 (0.49-0.85)	0.104
No	945/32817	599/18879	0.88 (0.76-1.02)	0.86 (0.74-1.00)	0.87 (0.75-1.01)	
**Parents diagnosed with hypertension**						
Yes	541/18077	386/11220	0.78 (0.65-0.94)	0.75 (0.62-0.91)	0.76 (0.63-0.92)	0.655
No	643/21986	405/12150	0.88 (0.73-1.05)	0.87 (0.72-1.04)	0.88 (0.73-1.04)	
**HF**						
**Sex**						
Male	517/17286	327/10051	0.79 (0.67-0.96)	0.77 (0.65-0.92)	0.76 (0.64-0.93)	0.779
Female	315/22777	188/13319	0.81 (0.63-1.05)	0.80 (0.62-1.04)	0.79 (0.61-1.03)	
**Ethnicity**						
White	794/38612	498/22417	0.77 (0.66-0.90)	0.76 (0.65-0.89)	0.75 (0.64-0.88)	0.232
Non-white	38/1451	17/953	1.22 (0.55-1.82)	1.15 (0.50-1.79)	1.10 (0.47-1.74)	
**Place of birth**						
England	746/34563	455/20018	0.84 (0.71-0.97)	0.83 (0.70-0.96)	0.82 (0.69-0.95)	0.078
Wales or Scotland	86/5500	60/3352	0.74 (0.46-1.18)	0.72 (0.44-1.17)	0.71 (0.43-1.16)	
**PRS for HF**						
Low	207/12836	117/7444	0.91 (0.66-1.23)	0.90 (0.65-1.22)	0.89 (0.64-1.21)	0.152
Medium	259/13194	182/7702	0.63 (0.49-0.82)	0.61 (0.47-0.80)	0.60 (0.46-0.79)	
High	329/12781	199/7504	0.85 (0.67-1.10)	0.84 (0.66-1.09)	0.83 (0.65-1.08)	
**Parents diagnosed with CVD**						
Yes	515/23425	295/13209	0.86 (0.71-1.04)	0.84 (0.69-1.03)	0.83 (0.68-1.02)	0.411
No	317/16638	220/10161	0.70 (0.55-0.89)	0.69 (0.54-0.88)	0.68 (0.53-0.87)	
**Parents diagnosed with diabetes**						
Yes	165/7246	110/4491	0.79 (0.57-1.11)	0.78 (0.56-1.10)	0.77 (0.55-1.09)	0.829
No	667/32817	405/18879	0.78 (0.66-0.93)	0.78 (0.65-0.92)	0.78 (0.65-0.92)	
**Parents diagnosed with hypertension**						
Yes	401/18077	227/11220	0.89 (0.70-1.10)	0.89 (0.70-1.09)	0.88 (0.69-1.09)	0.068
No	431/21986	288/12150	0.74 (0.61-0.88)	0.73 (0.60-0.87)	0.71 (0.58-0.86)	
**AF**						
**Sex**						
Male	1277/17286	708/10051	0.89 (0.79-1.00)	0.87 (0.77-0.98)	0.86 (0.76-0.97)	0.106
Female	774/22777	376/13319	0.91 (0.77-1.08)	0.90 (0.76-1.07)	0.89 (0.75-1.06)	
**Ethnicity**						
White	1972/38612	1044/22417	0.87 (0.79-0.97)	0.86 (0.78-0.96)	0.85 (0.77-0.95)	0.324
Non-white	79/1451	40/953	1.22 (0.74-2.05)	1.19 (0.71-2.03)	1.14 (0.66-1.96)	
**Place of birth**						
England	1799/34563	951/20018	0.88 (0.79-0.98)	0.87 (0.78-0.97)	0.86 (0.77-0.96)	0.090
Wales or Scotland	252/5500	133/3352	0.95 (0.69-1.25)	0.92 (0.67-1.23)	0.91 (0.66-1.22)	
**PRS for AF**						
Low	610/12839	306/7443	0.92 (0.75-1.11)	0.90 (0.74-1.09)	0.89 (0.73-1.08)	0.773
Medium	662/13193	346/7704	0.86 (0.72-1.03)	0.86 (0.72-1.02)	0.85 (0.71-1.02)	
High	710/12779	389/7503	0.88 (0.74-1.04)	0.87 (0.72-1.03)	0.86 (0.72-1.02)	
**Parents diagnosed with CVD**						
Yes	1255/23425	634/13209	0.93 (0.80-1.05)	0.91 (0.79-1.03)	0.90 (0.78-1.02)	0.791
No	796/16638	450/10161	0.81 (0.69-0.95)	0.79 (0.68-0.93)	0.79 (0.67-0.93)	

**Parents diagnosed with diabetes**						
Yes	390/7246	228/4491	0.86 (0.68-1.08)	0.83 (0.65-1.06)	0.81 (0.64-1.03)	0.610
No	1661/32817	856/18879	0.89 (0.79-0.99)	0.88 (0.78-0.98)	0.87 (0.77-0.97)	
**Parents diagnosed with hypertension**						
Yes	939/18077	524/11220	0.92 (0.79-1.06)	0.91 (0.79-1.05)	0.90 (0.77-1.04)	0.904
No	1112/21986	560/12150	0.85 (0.73-0.97)	0.84 (0.73-0.96)	0.84 (0.72-0.96)	
**Stroke**						
**Sex**						
Male	370/17286	228/10051	0.83 (0.66-1.04)	0.82 (0.65-1.03)	0.81 (0.64-1.02)	0.544
Female	273/22777	178/13319	0.81 (0.64-1.05)	0.80 (0.63-1.05)	0.79 (0.62-1.04)	
**Ethnicity**						
White	613/38612	392/22417	0.82 (0.69-0.98)	0.80 (0.67-0.95)	0.79 (0.66-0.95)	0.142
Non-white	30/1451	14/953	1.15 (0.49-2.78)	1.12 (0.46-2.76)	1.09 (0.43-2.74)	
**Place of birth**						
England	551/34563	354/20018	0.80 (0.67-0.97)	0.79 (0.66-0.96)	0.78 (0.65-0.95)	0.148
Wales or Scotland	92/5500	52/3352	0.92 (0.58-1.50)	0.89 (0.55-1.47)	0.87 (0.53-1.44)	
**PRS for stroke**						
Low	190/12835	104/7445	0.91 (0.65-1.27)	0.90 (0.64-1.26)	0.89 (0.63-1.25)	0.198
Medium	195/13194	120/7700	0.90 (0.65-1.24)	0.89 (0.65-1.23)	0.89 (0.64-1.23)	
High	241/12782	163/7505	0.75 (0.56-0.99)	0.74 (0.55-0.98)	0.73 (0.54-0.97)	
**Parents diagnosed with CVD**						
Yes	397/23425	250/13209	0.77 (0.62-0.95)	0.76 (0.61-0.94)	0.75 (0.60-0.93)	0.571
No	246/16638	156/10161	0.90 (0.68-1.20)	0.89 (0.67-1.19)	0.89 (0.67-1.19)	
**Parents diagnosed with diabetes**						
Yes	115/7246	70/4491	0.86 (0.58-1.28)	0.83 (0.55-1.25)	0.81 (0.53-1.23)	0.508
No	528/32817	336/18879	0.81 (0.67-0.98)	0.80 (0.66-0.97)	0.79 (0.65-0.96)	
**Parents diagnosed with hypertension**						
Yes	292/18077	187/11220	0.83 (0.65-1.07)	0.82 (0.64-1.06)	0.81 (0.63-1.05)	0.622
No	351/21986	219/12150	0.81 (0.64-1.02)	0.79 (0.62-1.01)	0.79 (0.62-1.00)	
**CVD mortality**						
**Sex**						
Male	350/17286	206/10051	0.91 (0.72-1.16)	0.88 (0.69-1.12)	0.87 (0.68-1.11)	0.597
Female	170/22777	105/13319	0.74 (0.53-1.05)	0.73 (0.52-1.03)	0.72 (0.51-1.02)	
**Ethnicity**						
White	498/38612	297/22417	0.83 (0.69-1.02)	0.81 (0.67-1.00)	0.80 (0.66-0.99)	0.698
Non-white	22/1451	14/953	1.20 (0.48-3.20)	1.16 (0.45-3.06)	1.13 (0.42-3.01)	
**Place of birth**						
England	454/34563	264/20018	0.88 (0.72-1.08)	0.87 (0.70-1.07)	0.85 (0.68-1.05)	0.418
Wales or Scotland	66/5500	47/3352	0.79 (0.48-1.40)	0.75 (0.43-1.32)	0.74 (0.42-1.30)	
**PRS for CVD**						
Low	144/12840	72/7441	0.85 (0.58-1.26)	0.83 (0.56-1.24)	0.82 (0.55-1.23)	0.431
Medium	155/13189	99/7707	0.80 (0.56-1.13)	0.78 (0.54-1.11)	0.76 (0.52-1.09)	
High	203/12782	120/7502	0.97 (0.70-1.34)	0.96 (0.69-1.32)	0.95 (0.68-1.31)	
**Parents diagnosed with CVD**						
Yes	328/23425	192/13209	0.82 (0.65-1.05)	0.80 (0.63-1.04)	0.79 (0.62-1.03)	0.821

No	192/16638	119/10161	0.89 (0.64-1.22)	0.87 (0.62-1.21)	0.87 (0.62-1.20)	
**Parents diagnosed with diabetes**						
Yes	86/7246	62/4491	0.80 (0.51-1.30)	0.79 (0.50-1.25)	0.76 (0.47-1.23)	0.374
No	434/32817	249/18879	0.85 (0.69-1.06)	0.83 (0.67-1.04)	0.82 (0.66-1.03)	
**Parents diagnosed with hypertension**						
Yes	231/18077	134/11220	0.89 (0.66-1.21)	0.87 (0.64-1.18)	0.86 (0.63-1.17)	0.369
No	289/21986	177/12150	0.82 (0.63-1.06)	0.80 (0.62-1.05)	0.79 (0.61-1.04)	

Model 1 and Model 2 were Cox proportional hazard model. In model 1, we adjusted for age and sex. Model 2 included age, sex, race, birth location, calendar month of birth, real food prices (adjusted for the consumer price index), parental disease history (CVD, diabetes, hypertension), genetic risk score for each outcome, maternal smoking around birth, whether breastfed as a baby and survey year. Model 3 adjusted for terms in Model 2 and used parametric hazard models based on the Gompertz distribution. CVD = cardiovascular disease; MI = myocardial infarction; HF = heart failure; AF = atrial fibrillation; PRS = polygenic risk score.

Supplementary Table E. The effect of rationing on age of disease onset by duration of exposure to rationing.

	**Not Rationed**	**In utero**	**In utero + (0, 1] year**	**In utero + (1, 2] year**
**CVD**				
Age of onset (95%CI)	61.67 (61.49, 61.86)	62.66 (62.39, 62.92)	63.22 (63.01, 63.44)	64.2 (63.99, 64.42)
Delay in age of onset (years) (95% CI)	**Reference**	0.98 (0.66, 1.3)	1.55 (1.27, 1.83)	2.53 (2.25, 2.81)
**MI**				
Age of onset (95%CI)	61.93 (61.64, 62.21)	62.75 (62.32, 63.17)	63.43 (63.08, 63.78)	64.6 (64.24, 64.97)
Delay in age of onset (years) (95% CI)	**Reference**	0.82 (0.31, 1.33)	1.5 (1.05, 1.95)	2.67 (2.21, 3.13)
**HF**				
Age of onset (95%CI)	62.73 (62.39, 63.08)	63.91 (63.4, 64.41)	64.49 (64.09, 64.89)	65.69 (65.29, 66.1)
Delay in age of onset (years) (95% CI)	**Reference**	1.17 (0.56, 1.78)	1.75 (1.22, 2.28)	2.96 (2.43, 3.49)
**AF**				
Age of onset (95%CI)	62.15 (61.91, 62.39)	63.09 (62.75, 63.43)	64.01 (63.72, 64.29)	64.88 (64.61, 65.15)
Delay in age of onset (years) (95% CI)	**Reference**	0.95 (0.53, 1.36)	1.86 (1.49, 2.23)	2.74 (2.38, 3.1)
**Stroke**				
Age of onset (95%CI)	62.26 (61.85, 62.68)	63.13 (62.54, 63.72)	63.83 (63.34, 64.32)	64.57 (64.03, 65.11)
Delay in age of onset (years) (95% CI)	**Reference**	0.86 (0.15, 1.58)	1.57 (0.93, 2.2)	2.31 (1.63, 2.98)
**CVD mortality**				
Age of onset (95%CI)	63.11 (62.69, 63.54)	63.19 (62.53, 63.85)	64.63 (64.13, 65.14)	65.75 (65.23, 66.27)
Delay in age of onset (years) (95% CI)	**Reference**	0.08 (-0.7, 0.86)	1.52 (0.87, 2.17)	2.64 (1.97, 3.31)

Time-to-event models assume Gompertz distribution were used. CVD = cardiovascular disease; MI = myocardial infarction; HF = heart failure; AF = atrial fibrillation.

Supplementary Table F. Hazard ratios for cardiovascular outcomes across early-life sugar rationing exposure categories under the model including later-life lifestyle and health factors.

	Not Rationed	In utero	In utero + (0, 1] year	In utero + (1, 2] year	P for trend
**CVD**	Reference	0.90 (0.83-0.98)	0.87 (0.79-0.95)	0.80 (0.72-0.89)	<0.001
**MI**	Reference	0.85 (0.74-0.98)	0.82 (0.71-0.96)	0.76 (0.64-0.91)	0.001
**HF**	Reference	0.79 (0.67-0.95)	0.80 (0.67-0.96)	0.76 (0.61-0.96)	0.029
**AF**	Reference	0.93 (0.83-1.04)	0.83 (0.73-0.94)	0.80 (0.69-0.92)	<0.001
**Stroke**	Reference	0.81 (0.67-0.99)	0.78 (0.63-0.97)	0.69 (0.54-0.89)	0.002
**CVD mortality**	Reference	0.82 (0.65-1.03)	0.82 (0.65-1.03)	0.73 (0.54-0.98)	0.061

Model included age, sex, race, birth location, calendar month of birth, real food prices (adjusted for the consumer price index), genetic risk score for cardiovascular outcomes, parental disease history (CVD, diabetes, hypertension), maternal smoking around birth, whether breastfed as a baby, survey year, physical activity, smoking, alcohol intake, comorbidities, and social fat intake. Model 4 includes all covariates from Model 2, as well as later-life factors (physical activity, smoking, alcohol intake, comorbidities, and social fat intake). This model is presented as a robustness check and is not used for causal interpretation. CVD = cardiovascular disease; MI = myocardial infarction; HF = heart failure; AF = atrial fibrillation

Supplementary Table G. Baseline characteristics of individuals born outside the UK who have not experienced sugar rationing, compared with participants born in the UK.

	**Participants born outside the UK**	**Participants born in the UK**		**P value***	**P value#**
		**Rationed**	**Not Rationed**		
**No. of participants**	2864	40063	23370		
**Age at entry, mean (SD), years**	54.8 (1.6)	55.4 (1.2)	53.2 (1.0)	0.112	0.003
**Women**	1638 (57.2)	22777 (56.9)	13319 (57.0)	0.865	0.927
**Birth month**					
Mar 1-May 31	684 (23.9)	11742 (29.3)	4857 (20.8)	<0.001	<0.001
Jun 1-Aug 31	622 (21.7)	8608 (21.5)	5720 (24.5)		
Sep 1-Nov 30	770 (26.9)	9082 (22.7)	6340 (27.1)		
Dec 1-Feb 28	788 (27.5)	10631 (26.5)	6453 (27.6)		
**White, %**	2755 (96.2)	38612 (96.4)	22417 (95.9)	0.955	0.931
**Education, %**					
Below A levels	733 (25.6)	16647 (41.6)	9480 (40.6)	<0.001	<0.001
A levels (high school)	854 (29.8)	12287 (30.7)	7658 (32.8)		
College/university	874 (30.5)	4960 (12.4)	2858 (12.2)		
Professional/other	403 (14.1)	6169 (15.4)	3374 (14.4)		
**Household income, £**					
<18,000	704 (24.6)	9772 (24.4)	5114 (21.9)	0.876	0.010
18,000-30,999	451 (15.7)	7666 (19.1)	4119 (17.6)		
31,000-51,999	702 (24.5)	10551 (26.3)	6241 (26.7)		
52,000-100,000	710 (24.8)	9394 (23.4)	6079 (26.0)		
>100,000	297 (10.4)	2680 (6.7)	1817 (7.8)		
**Townsend deprivation index, mean (SD)**	-0.44 (3.4)	-1.5 (3.0)	-1.4 (3.0)	<0.001	<0.001
**Smoking status, %**					
Never	1431 (50.0)	22406 (55.9)	13365 (57.2)	<0.001	<0.001
Former	1087 (38.0)	13381 (33.4)	7250 (31.0)		
Current	346 (12.1)	4276 (10.7)	2755 (11.8)		
**Alcohol consumption frequency, times/wk**					
< 3	1633 (57.0)	21464 (53.6)	12670 (54.2)	0.054	0.072
>=3	1231 (43.0)	18599 (46.4)	10700 (45.8)		
**BMI, mean (SD), kg/m2**	27.4 (5.1)	27.5 (5.0)	27.4 (5.0)	0.263	0.684
**Summed MET minutes per week for all activity, IQR**	1863 (892.5, 3342.4)	1806 (795-3532)	1794 (807-3492)	0.026	<0.001
**Personal medical condition**					
Hypertension	1552 (54.2)	22627 (56.5)	12688 (54.3)	0.215	0.968
High cholesterol	581 (20.3)	7388 (18.4)	3890 (16.6)	0.045	0.008
Diabetes	189 (6.6)	2127 (5.3)	1161 (5.0)	0.006	0.002
Digestive disease	34 (1.2)	337 (0.8)	176 (0.8)	0.062	0.020
Kidney disease	94 (3.3)	1086 (2.7)	556 (2.4)	0.090	0.056
Liver disease	135 (4.7)	1409 (3.5)	767 (3.3)	0.002	<0.001
**Parents' condition**					
†Parents diagnosed with CVD	1712 (59.8)	23425 (58.5)	13209 (56.5)	0.495	0.128
†Parents diagnosed with diabetes	461 (16.1)	7246 (18.1)	4491 (19.2)	0.025	0.003
†Parents diagnosed with hypertension	1426 (49.8)	18077 (45.1)	11220 (48.0)	0.003	0.397
Parents still alive	421 (14.7)	5373 (13.4)	4697 (20.1)	0.095	<0.001
**Birth weight, mean (SD), kg**	3.3 (0.6)	3.3 (0.5)	3.3 (0.5)	0.567	0.398
**Maternal smoking around birth**	914 (31.9)	12216 (30.5)	7138 (30.5)	0.255	0.198
**Breastfed as a baby**	1618 (56.5)	23564 (58.8)	13601 (58.2)	0.216	0.251

Data are presented as No. (%), mean (SD) or median (IQR). * Comparison between participants born outside the UK and participants born in the UK (Rationed). # Comparison between participants born outside the UK and participants born in the UK (Not Rationed). † We applied the Mantel-Haenszel Chi-Square test to adjust for whether the participants' parents were alive during the survey. We have applied the Bonferroni correction to the p-values, and the adjusted significance threshold for the tests is 0.05/25 = 0.002. BMI = body mass index; MET: metabolic equivalent of task; IQR: interquartile range; CVD: cardiovascular disease.

Supplementary Table H. Baseline characteristics of participants in external validation cohort (ELSA).

	**Overall**	**Non-rationed**	**Rationed**	**P**
**Participants**	1694	759	935	
**Age (mean (SD))**	52.2 (1.7)	50.8 (1.5)	53.3 (0.9)	<0.001
**Sex (%)**				
Female	960 (56.7)	432 (56.9)	528 (56.5)	0.955
Male	734 (43.3)	327 (43.1)	407 (43.5)	
**Race (%)**				
Non-white	63 (3.7)	27 (3.6)	36 (3.8)	0.86
White	1631 (96.3)	732 (96.4)	899 (96.2)	
**Education (%)**				
Below high school	385 (22.7)	170 (22.4)	215 (23.0)	0.131
College or above	801 (47.3)	352 (46.4)	449 (48.0)	
High school	392 (23.1)	193 (25.4)	199 (21.3)	
Other	116 (6.8)	44 (5.8)	72 (7.7)	
**Family wealth, pounds (mean (SD))**	264791.7 (351849.1)	264180.2 (365393.7)	265288.1 (340654.8)	0.949
**Marital status (%)**				
Married or partnered	1667 (98.4)	753 (99.2)	914 (97.8)	0.001
Never married	2 (0.1)	1 (0.1)	1 (0.1)	
Separated/divorced/Widowed	25 (1.5)	5 (0.7)	20 (2.1)	
**Physical activity (%)**				
> 1 per week	465 (27.4)	192 (25.3)	273 (29.2)	0.003
1 per week	121 (7.1)	44 (5.8)	77 (8.2)	
1-3 per mon	169 (10.0)	66 (8.7)	103 (11.0)	
hardly ever or never	939 (55.4)	457 (60.2)	482 (51.6)	
**Smoking status (%)**				
Ever smokers	1029 (60.7)	445 (58.6)	584 (62.5)	0.120
Never smokers	665 (39.3)	314 (41.4)	351 (37.5)	
**Drinking status (%)**				
Drank in past 12 months	1596 (94.2)	714 (94.1)	882 (94.3)	0.902
Did not drink in past 12 months	98 (5.8)	45 (5.9)	53 (5.7)	
**Hypertension (%)**				
No	1318 (77.8)	590 (77.7)	728 (77.9)	0.882
Yes	376 (22.2)	169 (22.3)	207 (22.1)	
**Diabetes (%)**				
No	1651 (97.5)	738 (97.2)	913 (97.6)	0.914
Yes	43 (2.5)	21 (2.8)	22 (2.4)	

Data are presented as No. (%) or mean (SD). * P values were obtained from either a chi-square test or a Mann-Whitney U test comparing difference between rationed and not rationed group.

Supplementary Table I. Baseline data for the contemporaneous control group (HRS), including individuals without exposure to sugar rationing.

	**Overall**	**Born between July 1954 and March 1956**	**Born between October 1951 and June 1954**	**P**
**Participants**	1763	916	847	
**Age (mean (SD))**	51.7 (1.7)	50.5 (1.3)	53.0 (1.0)	<0.001
**Sex (%)**				
Female	992 (56.3)	512 (55.9)	480 (56.7)	0.893
Male	771 (43.7)	404 (44.1)	367 (43.3)	
**Race (%)**				
Non-white	71 (4.0)	40 (4.4)	31 (3.7)	0.546
White	1692 (96.0)	876 (95.6)	816 (96.3)	
**Education (%)**				
Below high school	307 (17.4)	159 (17.4)	148 (17.5)	0.987
College or above	922 (52.3)	478 (52.2)	444 (52.4)	
High school	534 (30.3)	279 (30.5)	255 (30.1)	
**Marital status (%)**				
Married or partnered	1719 (97.5)	895 (97.7)	824 (97.3)	0.486
Never married	1 (0.1)	1 (0.1)	0 (0.0)	
Separated/divorced/Widowed	43 (2.4)	20 (2.2)	23 (2.7)	
**Total wealth, pounds (mean (SD))**	215857.9 (399855.7)	209417.8 (378205.2)	222293.7 (420407.7)	0.378
**Smoking status (%)**				
Ever smokers	1031 (58.5)	504 (55.0)	527 (62.2)	0.003
Never smokers	732 (41.5)	412 (45.0)	320 (37.8)	
**Drinking status (%)**				
Drank in past 3 months	1039 (58.9)	559 (61.0)	480 (56.7)	0.07
Did not drink in past 3 months	724 (41.1)	357 (39.0)	367 (43.3)	
**Physical activity (%)**				
Vigorous activity 3+/wk	778 (44.1)	465 (50.8)	313 (37.0)	<0.001
Vigorous activity less than 3/wk	985 (55.9)	451 (49.2)	534 (63.0)	
**Hypertension (%)**				
No	1439 (81.6)	751 (82.0)	688 (81.2)	0.924
Yes	324 (18.4)	165 (18.0)	159 (18.8)	
**Diabetes (%)**				
No	1726 (97.9)	895 (97.7)	831 (98.1)	0.978
Yes	37 (2.1)	21 (2.3)	16 (1.9)	

Data are presented as No. (%) or mean (SD). * P values were obtained from either a chi-square test or a Mann-Whitney U test comparing difference between rationed and not rationed group.

Supplementary Table J. Cardiovascular outcomes among non-UK born participants

	**Not Rationed**	**Rationed**
**CVD**		
Total cases/Total sample size	89/1081	157/1783
Model 1	Reference	1.02 (0.86-1.18)
Model 2	Reference	0.99 (0.83-1.15)
**MI**		
Total cases/Total sample size	44/1081	90/1783
Model 1	Reference	1.04 (0.83-1.25)
Model 2	Reference	1.01 (0.80-1.22)
**HF**		
Total cases/Total sample size	30/1081	62/1783
Model 1	Reference	1.00 (0.77-1.24)
Model 2	Reference	0.98 (0.75-1.21)
**AF**		
Total cases/Total sample size	57/1081	112/1783
Model 1	Reference	1.03 (0.86-1.20)
Model 2	Reference	1.01 (0.84-1.18)
**Stroke**		
Total cases/Total sample size	27/1081	50/1783
Model 1	Reference	1.06 (0.82-1.30)
Model 2	Reference	1.03 (0.79-1.27)
**CVD mortality**		
Total cases/Total sample size	21/1081	43/1783
Model 1	Reference	1.03 (0.78-1.28)
Model 2	Reference	0.99 (0.74-1.24)

Model 1 and Model 2 were Cox proportional hazard model. Model 1 adjusted for age and sex. Model 2 included age, sex, race, calendar month of birth and survey year. CVD = cardiovascular disease; MI = myocardial infarction; HF = heart failure; AF = atrial fibrillation.

Supplementary Table K. Odds of low left ventricular ejection fraction (<50%) by sugar rationing exposure group

Group	Event (n, %)	Total (n)	Model 1 OR (95% CI)	P-value	Model 2 OR (95% CI)	P-value
**Low LVEF (<50%)**						
Not Rationed	439 (17.5%)	2509	**Ref.**	—	**Ref.**	—
Rationed	599 (14.2%)	4208	0.78 (0.67-0.93)	0.004	0.81 (0.69-0.95)	0.009

*Values are presented as n (%). OR = odds ratio from logistic regression. Model 1 adjusted for age and sex. Model 2 included age, sex, race, birth location, calendar month of birth, real food prices (adjusted for the consumer price index), parental disease history (CVD, diabetes, hypertension), maternal smoking around birth, whether breastfed as a baby and survey year. LVEF: left ventricular ejection fraction.

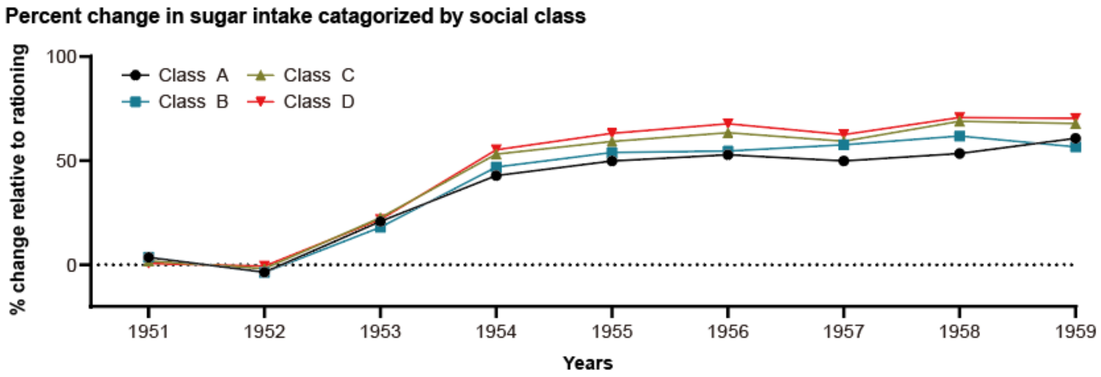

Supplementary Figure A. Annual variation of sugar intake by social class during the study period. Panels A, B, and C use the average consumption between 1950 Q1 and 1953 Q3 as the reference value. Panel D uses the average of 1951 and 1952 as the reference. Panels A, B, and D show percentage changes. Panel C shows raw changes.

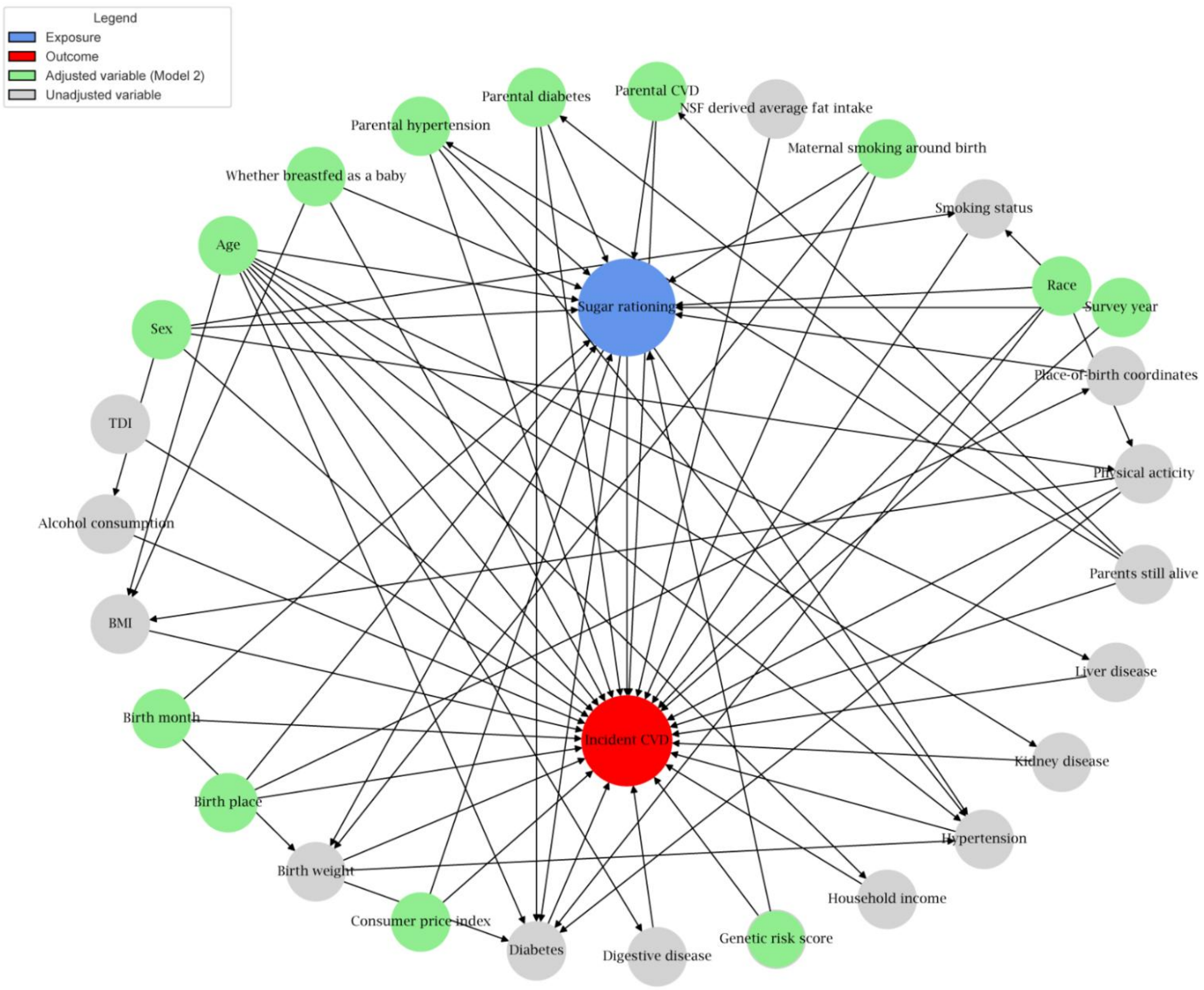

Supplementary Figure B. Directed Acyclic Graph. The directed acyclic graph above was generated via the R package 'dagitty'.[20] CVD: Cardiovascular disease; NSF: National Food Survey; TDI = Townsend deprivation index; BMI = Body Mass Index.

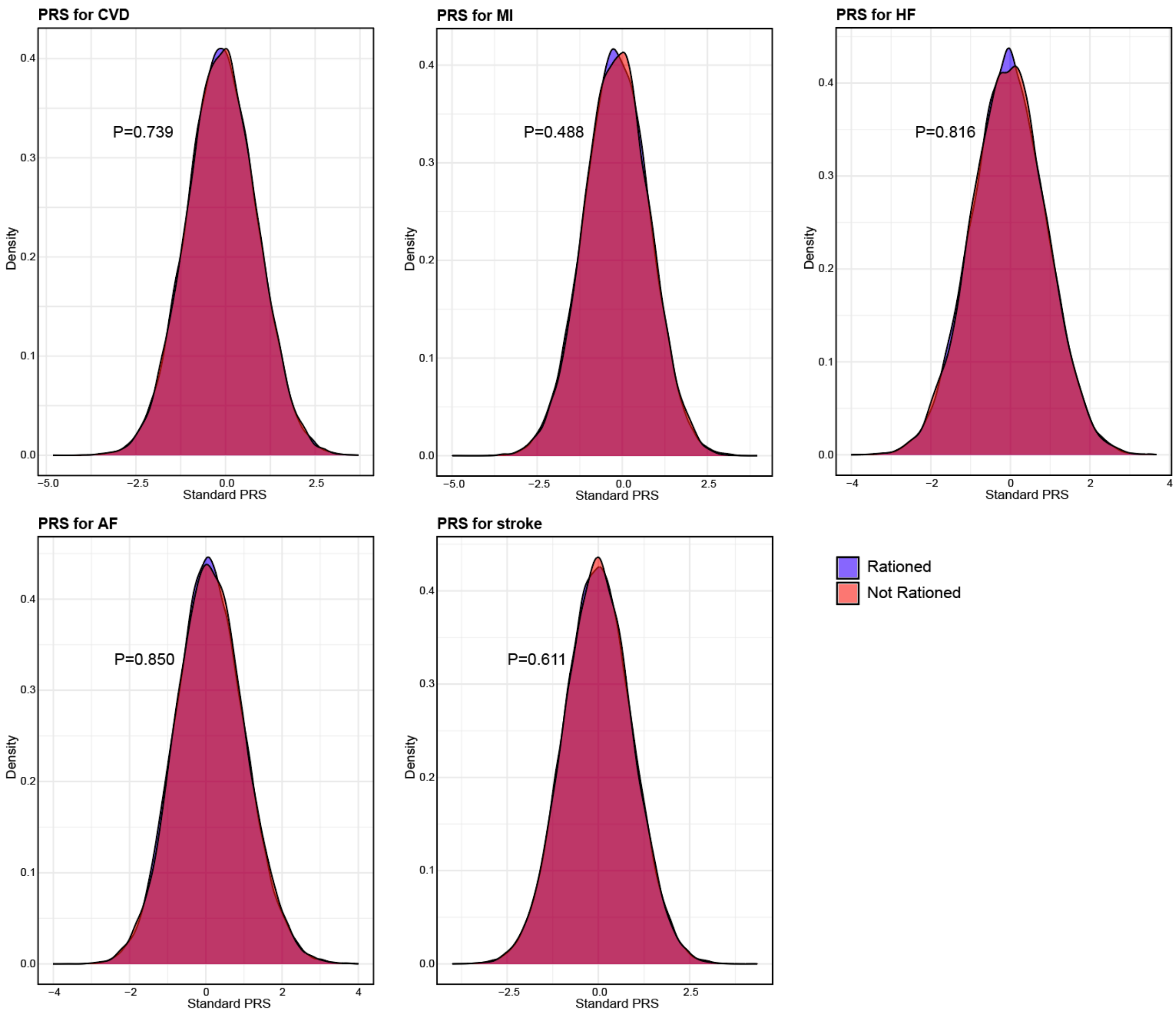

Supplementary Figure C. Comparative density distributions of PRS (CVD, MI, HF, AF, stroke) between rationed and not rationed groups. The difference between the two groups' PRS was assessed using a t-test. CVD = cardiovascular disease; MI = myocardial infarction; HF = heart failure; AF = atrial fibrillation; PRS = polygenic risk score.

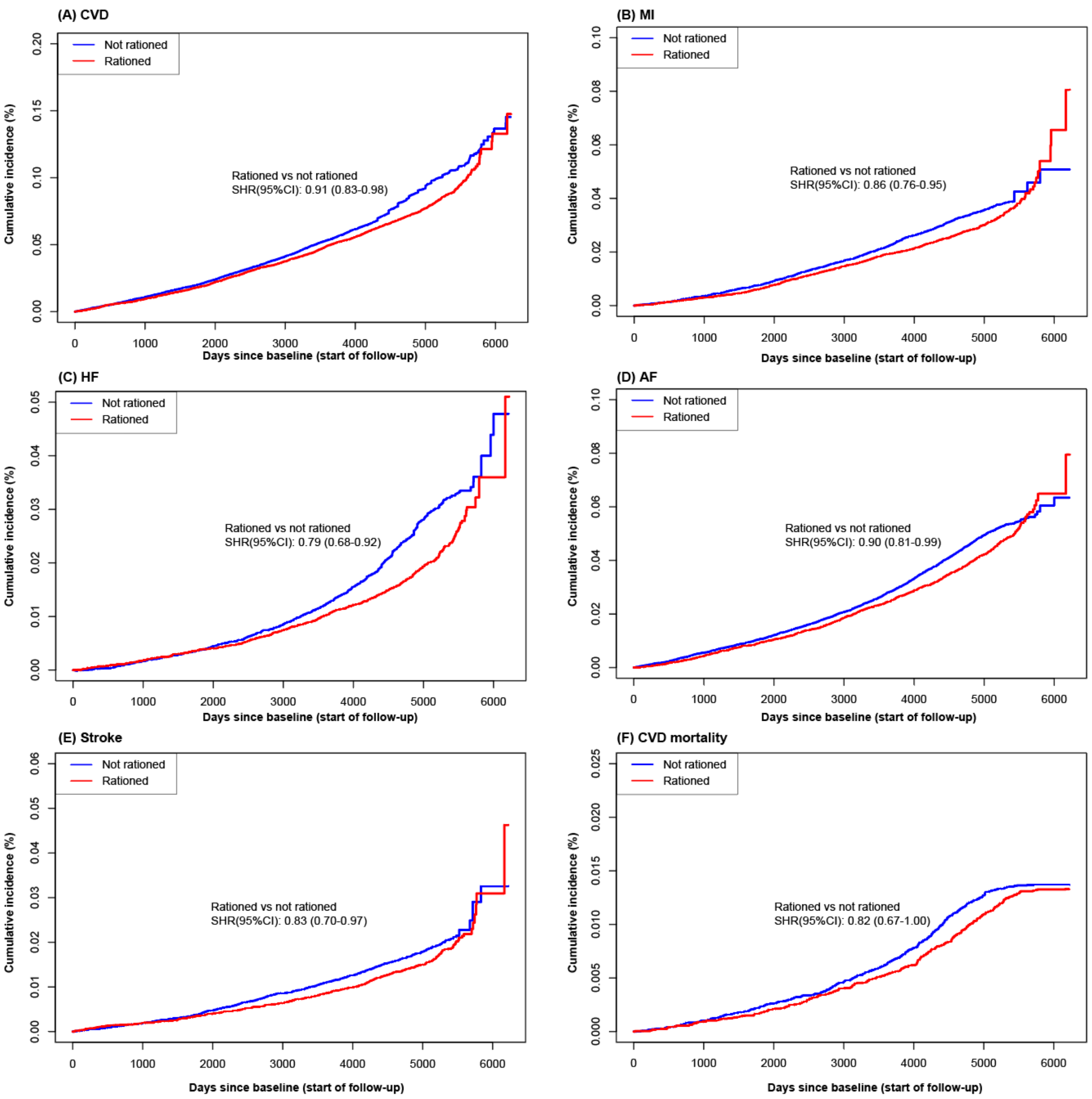

Supplementary Figure D. Cumulative incidence of cardiovascular outcomes between individuals exposed to sugar rationing and never-rationed adults. A Fine and Gray model was used to adjust for competing risks, with the competing events being non-CVD mortality. There were 2697 competing risk events recorded for CVD, 3139 for MI, 3037 for HF, 2976 for AF, 3181 for stroke, and 2624 for CVD mortality. CI = confidential interval; SHR, sub-distribution hazard ratio. Model was adjusted for covariates included in the model 2. SHR values (95% CI) represent the relative risk of each outcome between the rationed and non-rationed groups. CVD = cardiovascular disease; MI = myocardial infarction; HF = heart failure; AF = atrial fibrillation.

(A)

	Not Rationed	In utero	In utero + (0, 1] year	In utero + (1, 2] year	P for trend
Total death/ Total sample size	1308/23370	546/10466	766/14685	770/14912	
Model 1 HR (95%CI)	Reference	0.89 (0.79-0.98)	0.85 (0.75-0.94)	0.80 (0.70-0.92)	<0.001
Model 2 HR (95%CI)	Reference	0.86 (0.76-0.95)	0.82 (0.73-0.92)	0.77 (0.66-0.90)	<0.001

(B)

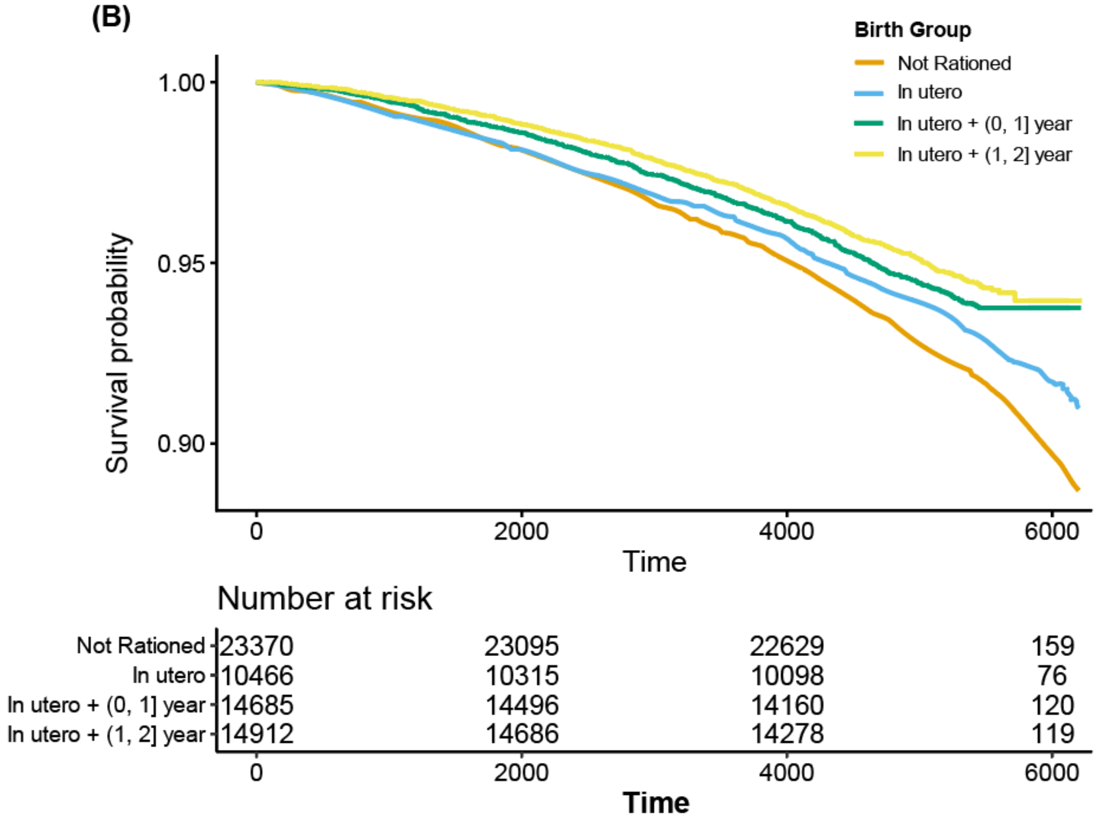

Supplementary Figure E. Association between sugar rationing exposure and all-cause mortality. Model 1 adjusted for age and sex. Model 2 included age, sex, race, birth location, calendar month of birth, real food prices (adjusted for the consumer price index), parental disease history (CVD, diabetes, hypertension), maternal smoking around birth, whether breastfed as a baby and survey year. (B) presents the survival curves derived from Cox regression (Model 2), adjusted for covariates, using the ggadjustedcurves function in R.[21] HR = hazard ratio; CI = confidence interval.

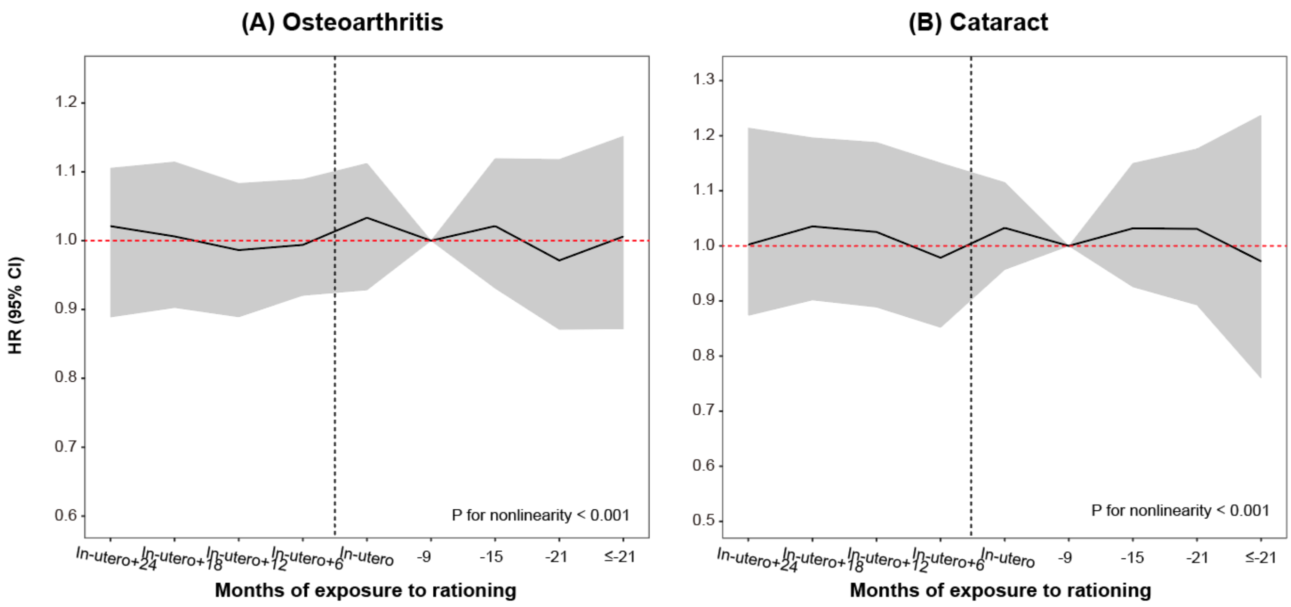

Supplementary Figure F. Hazard ratios for placebo outcomes (osteoarthritis [n=9,815] and cataract [n=4,187]) by various levels of rationing exposure. Parametric hazard models based on the Gompertz distribution were used. Penalized splines were adjusted for age, sex, race, birth location, calendar month of birth, real food prices (adjusted for the consumer price index), parental disease history (CVD, diabetes, hypertension), maternal smoking around birth, whether breastfed as a baby and survey year. The shaded area represents the 95% confidence interval. The vertical dashed black line indicates the end of sugar rationing.

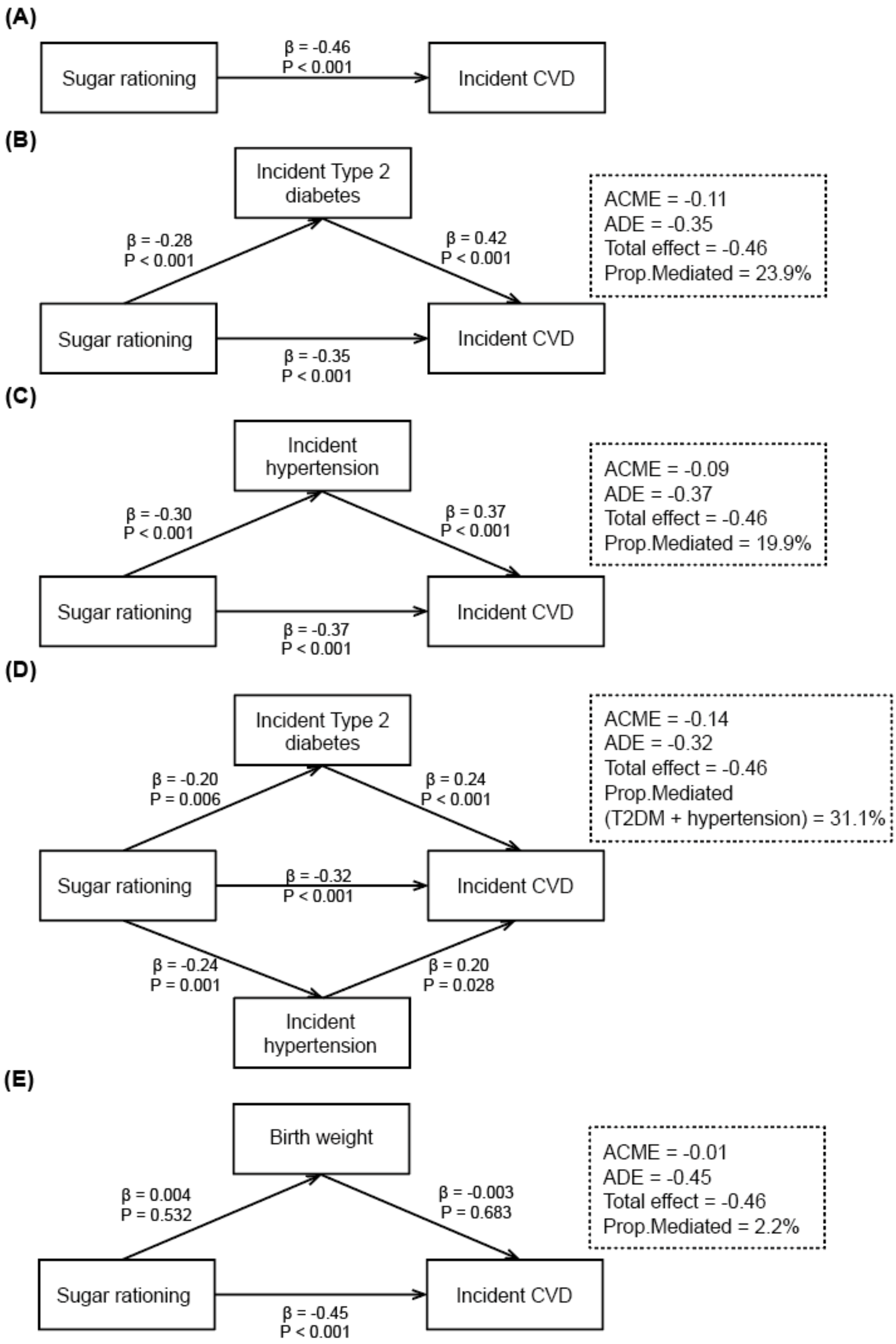

Supplementary Figure G. The standard mediation analysis investigating the proportion mediated by diabetes, hypertension, and birth weight in the relationship between sugar rationing and cardiovascular disease. We applied a standard mediation analysis (Kenny and Baron 4 step analysis).[16] Covariates in Model 2 were adjusted for in the linear regression model. CVD = cardiovascular disease; ACME = average causal mediated effect; ADE = average direct effect; T2DM = type 2 diabetes mellitus; Prop. Mediated = proportion mediated.

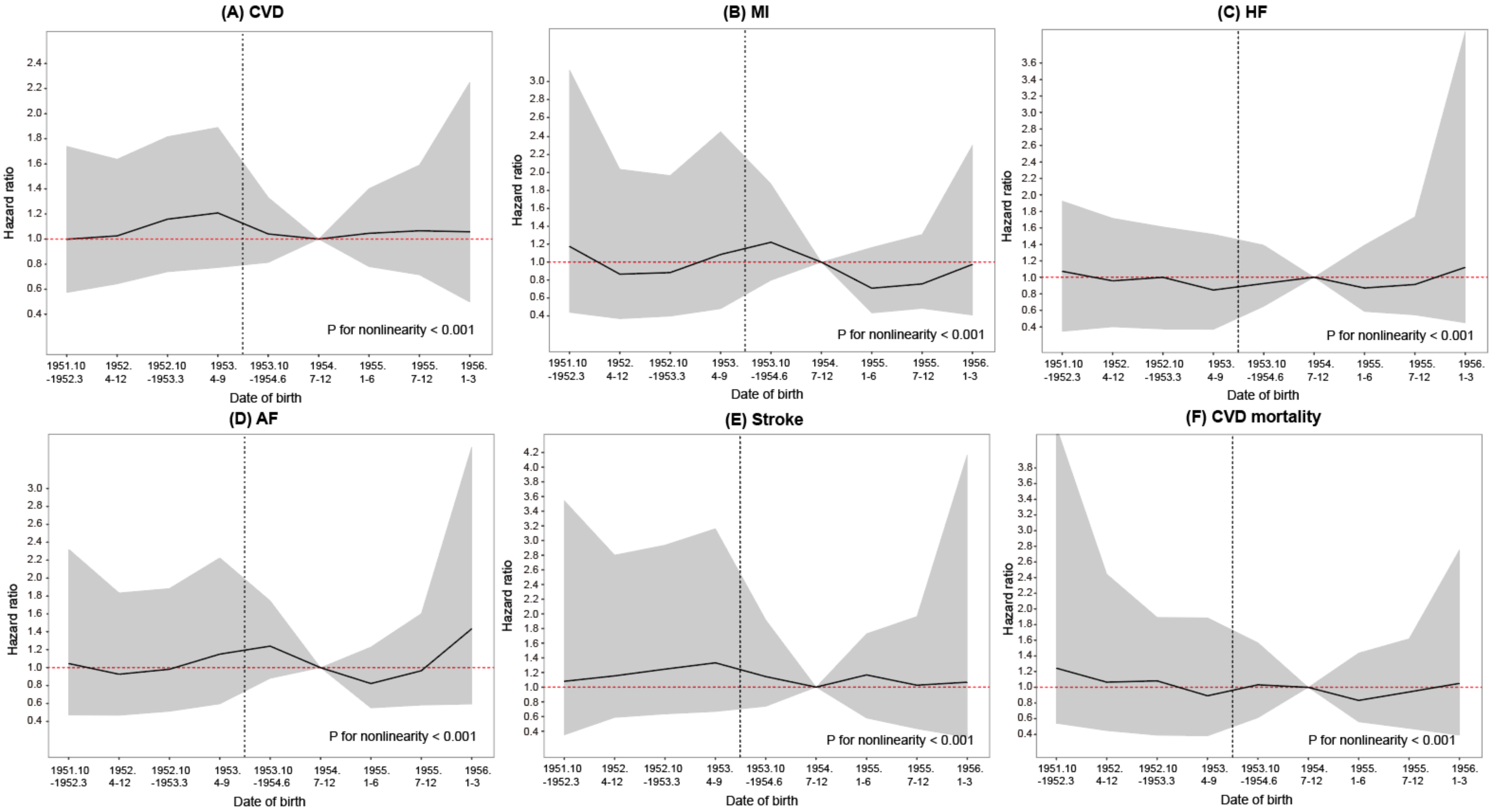

Supplementary Figure H. Hazard ratios for different cardiovascular outcomes by date of birth in individuals born outside of UK and have not experienced sugar rationing (n=2864). After frequency matching with the UK-born group on age, sex, and race, we included 2,864 participants from the contemporaneous control group who met the matching criteria. Parametric hazard models based on the Gompertz distribution were used. Model included age, sex, race, calendar month of birth and survey year. The shaded area represents the 95% confidence interval. The vertical dashed black line indicates the end of sugar rationing. CVD = cardiovascular disease; MI = myocardial infarction; HF = heart failure; AF = atrial fibrillation.

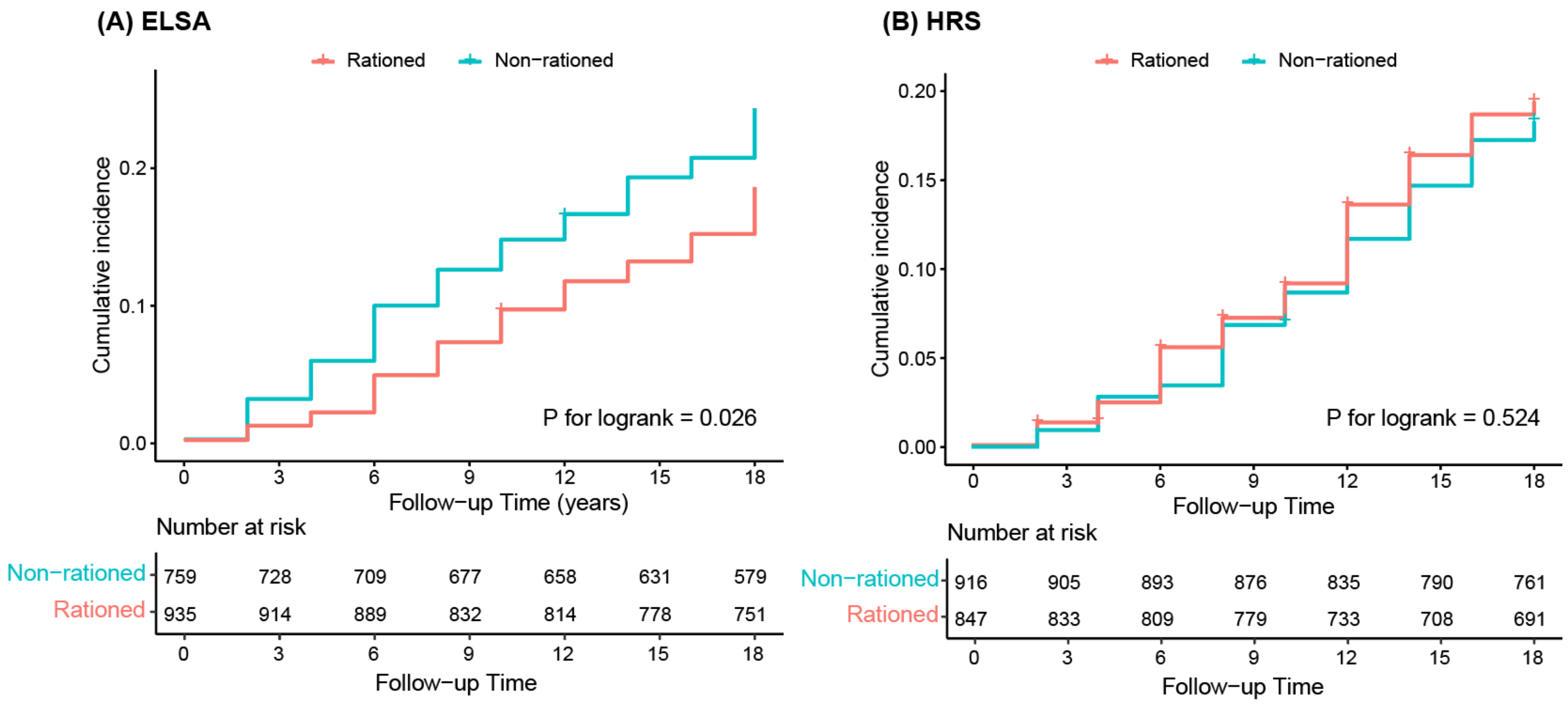

(C)

Cohort	Event (n)/Person-years in non-rationed participants (N)	Event (n)/Person-years in rationed participants (N)	Rationed vs not rationed-- Model 1 HR (95% CI)	P for model 1	Rationed vs not rationed-- Model 2 HR (95% CI)	P for model 2	Rationed vs not rationed-- Model 3 HR (95% CI)	P for Model 3
**ELSA**	179/12494	183/15132	0.88 (0.71-1.05)	0.083	0.80 (0.63-0.97)	0.027	0.81 (0.64-0.98)	0.035
**HRS**	153/15628	148/13990	1.06 (0.86-1.25)	0.613	1.00 (0.80-1.18)	0.741	1.02 (0.82-1.21)	0.722

Supplementary Figure I. Association between sugar rationing exposure and cumulative incidence of heart problem in ELSA and HRS. The HRs are derived by comparing participants born between October 1951 and June 1954 to those born between July 1954 and March 1956. Parametric hazard models based on the Gompertz distribution were used. Model 1 adjusts for age, sex, and race. Model 2 adjusts for model 1 plus education and marital status. Model 3 adjusts for model 2 plus survey year. HR = hazard ratio; CI = confidence interval.

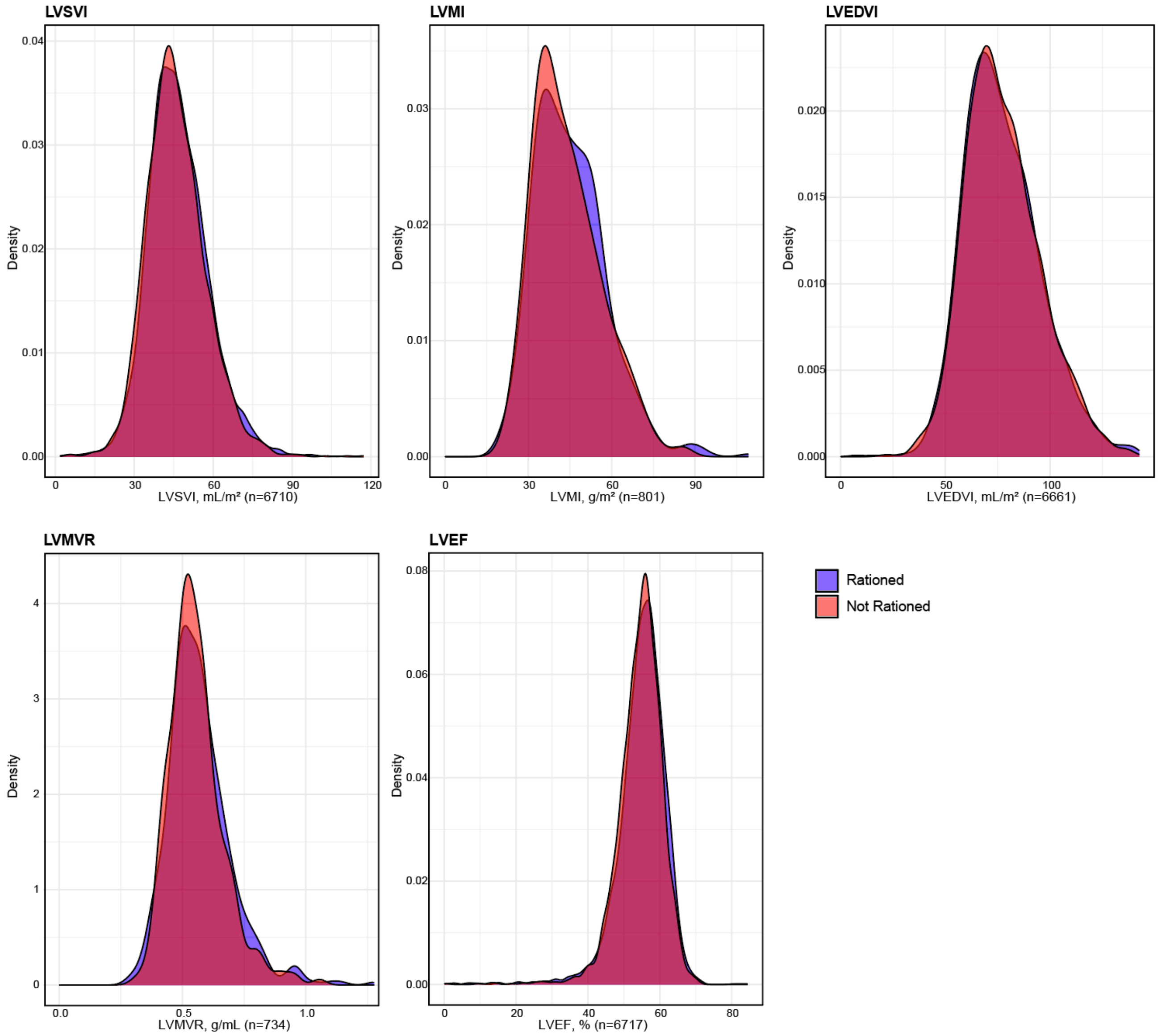

Supplementary Figure J. Comparative Density Distributions of Cardiac Metrics (LVSVI, LVMI, LVEDVI, LVMVR, LVEF) Between Rationed and Not Rationed Groups. LVSVI = left ventricular stroke volume index; LVMI: left ventricular mass index; LVEDVI: left ventricular end-diastolic volume index; LVMVR: left ventricular mass-to-volume ratio; LVEF: left ventricular ejection fraction.

Reference:

1. Gracner, T.; Boone, C.; Gertler, P. J., Exposure to sugar rationing in the first 1000 days of life protected against chronic disease. *Science* **2024,** *386* (6725), 1043-1048.
2. Jarman, B.; Townsend, P.; Carstairs, V., Deprivation indices. *BMJ* **1991,** *303* (6801), 523.
3. Howie, B. N.; Donnelly, P.; Marchini, J., A flexible and accurate genotype imputation method for the next generation of genome-wide association studies. *PLoS Genet.* **2009,** *5* (6), e1000529.
4. Ripatti, S.; Tikkanen, E.; Orho-Melander, M.; Havulinna, A. S.; Silander, K.; Sharma, A., et al., A multilocus genetic risk score for coronary heart disease: case-control and prospective cohort analyses. *The Lancet* **2010,** *376* (9750), 1393-1400.
5. Malik, R.; Chauhan, G.; Traylor, M.; Sargurupremraj, M.; Okada, Y.; Mishra, A., et al., Multiancestry genome-wide association study of 520,000 subjects identifies 32 loci associated with stroke and stroke subtypes. *Nat. Genet.* **2018,** *50* (4), 524-537.
6. Shah, S.; Henry, A.; Roselli, C.; Lin, H.; Sveinbjörnsson, G.; Fatemifar, G., et al., Genome-wide association and Mendelian randomisation analysis provide insights into the pathogenesis of heart failure. *Nat. Commun.* **2020,** *11* (1), 1-12.
7. Christophersen, I. E.; Rienstra, M.; Roselli, C.; Yin, X.; Geelhoed, B.; Barnard, J., et al., Large-scale analyses of common and rare variants identify 12 new loci associated with atrial fibrillation. *Nat. Genet.* **2017,** *49* (6), 946-952.
8. Dönertaş, H. M.; Fabian, D. K.; Fuentealba, M.; Partridge, L.; Thornton, J. M., Common genetic associations between age-related diseases. *Nature aging* **2021,** *1* (4), 400-412.
9. Littlejohns, T. J.; Holliday, J.; Gibson, L. M.; Garratt, S.; Oesingmann, N.; Alfaro-Almagro, F., et al., The UK Biobank imaging enhancement of 100,000 participants: rationale, data collection, management and future directions. *Nat. Commun.* **2020,** *11* (1), 2624.
10. Petersen, S. E.; Matthews, P. M.; Francis, J. M.; Robson, M. D.; Zemrak, F.; Boubertakh, R., et al., UK Biobank's cardiovascular magnetic resonance protocol. *J. Cardiovasc. Magn. Reson.* **2016,** *18* (1), 8.
11. Bai, W.; Sinclair, M.; Tarroni, G.; Oktay, O.; Rajchl, M.; Vaillant, G., et al., Automated cardiovascular magnetic resonance image analysis with fully convolutional networks. *J. Cardiovasc. Magn. Reson.* **2018,** *20* (1), 65.
12. Schulz-Menger, J.; Bluemke, D. A.; Bremerich, J.; Flamm, S. D.; Fogel, M. A.; Friedrich, M. G., et al., Standardized image interpretation and post-processing in cardiovascular magnetic resonance-2020 update: Society for Cardiovascular Magnetic Resonance (SCMR): Board of Trustees Task Force on Standardized Post-Processing. *J. Cardiovasc. Magn. Reson.* **2020,** *22* (1), 19.
13. Kleiber, M., Body size and metabolic rate. *Physiol. Rev.* **1947,** *27* (4), 511-541.
14. Bailey, B.; Briars, G., Estimating the surface area of the human body. *Stat. Med.* **1996,** *15* (13), 1325-1332.
15. Aung, N.; Wang, Q.; van Duijvenboden, S.; Burns, R.; Stoma, S.; Raisi-Estabragh, Z., et al., Association of longer leukocyte telomere length with cardiac size, function, and heart failure. *JAMA cardiology* **2023,** *8* (9), 808-815.
16. Baron, R. M.; Kenny, D. A., The moderator–mediator variable distinction in social psychological research: Conceptual, strategic, and statistical considerations. *J. Pers. Soc. Psychol.* **1986,** *51* (6), 1173.
17. Steptoe, A.; Breeze, E.; Banks, J.; Nazroo, J., Cohort profile: the English longitudinal study of ageing. *Int. J. Epidemiol.* **2013,** *42* (6), 1640-1648.
18. Sonnega, A.; Faul, J. D.; Ofstedal, M. B.; Langa, K. M.; Phillips, J. W.; Weir, D. R., Cohort profile:

the health and retirement study (HRS). *Int. J. Epidemiol.* **2014,** *43* (2), 576-585.
19. Van Buuren, S.; Groothuis-Oudshoorn, K., mice: Multivariate imputation by chained equations in R. *Journal of statistical software* **2011,** *45,* 1-67.
20. Textor, J.; Van der Zander, B.; Gilthorpe, M. S.; Liśkiewicz, M.; Ellison, G. T., Robust causal inference using directed acyclic graphs: the R package 'dagitty'. *Int. J. Epidemiol.* **2016,** *45* (6), 1887-1894.
21. Denz, R.; Timmesfeld, N., adjustedCurves: Estimating Confounder-Adjusted Survival Curves in R. *arXiv preprint arXiv:2402.15292* **2024**.
